# Molecular Engineering of Near-Infrared Light-Responsive BODIPY-Based Nanoparticles with Enhanced Photothermal and Photoacoustic Efficiencies for Cancer Theranostics

**DOI:** 10.7150/thno.34418

**Published:** 2019-07-09

**Authors:** Duyang Gao, Boyu Zhang, Yubin Liu, Dehong Hu, Zonghai Sheng, Xuanjun Zhang, Zhen Yuan

**Affiliations:** 1 Faculty of Health Sciences, University of Macau, Macau SAR, 999078, PR China; 2 Paul C. Lauterbur Research Center for Biomedical Imaging, Institute of Biomedical and Health Engineering, Shenzhen Key Laboratory of Ultrasound Imaging and Therapy, Shenzhen Institutes of Advanced Technology, Chinese Academy of Sciences, Shenzhen, 518055, China.; 3 College of Medical Laboratory, Dalian Medical University, No. 9 West Section LvShun South Road, Dalian 116044, P. R. China

**Keywords:** BODIPY molecular engineering, photoacoustic imaging, photothermal therapy, near-infrared absorption, theragnostic nanoparticles

## Abstract

**Background**: Engineering a single organic-molecule-based nanoparticle integrating precise diagnosis and effective therapy is of great significance for cancer treatment and future clinical applications but remains a great challenge. The goal of this study is to explore small organic molecule-based nanoparticles with high photothermal conversion efficiency for photoacoustic imaging-guided therapy.

**Methods**: Heptacyclic B, O-chelated BODIPY structure (namely Boca-BODIPY) with strong near-infrared (NIR) absorption was designed as a theranostic agent through simply molecular engineering, in which heavy atoms and alkyl chains were introduced to promote its application for tumor theranostics. The Boca-BODIPY molecules are further encapsulated in reduced bovine serum albumin (BSA) through self-assembly.

**Results**: The BSA-Boca-BODIPY exhibited excellent biocompatibility, extraordinary stability and high photothermal conversion efficiency up to 58.7%. The nanoparticles could dramatically enhance photoacoustic contrast of the tumor region, and the signal-to-noise ratio was increased about 14 times at 10 h post intravenous injection in 4T1 tumor-bearing mice. In addition, the nanoassemblies can efficiently convert laser energy (808 nm, 0.75 w cm^-2^, 5min) into hyperthermia for tumor ablation. Under the photoacoustic imaging-guided photothermal therapy (PTT), the 4T1 cancer cells were efficiently killed, no tumor recurrence and PTT-induced toxicity is observed.

**Conclusions**: Molecular engineering is a promising way to design organic-molecule-based nanoparticles for cancer theranostics. Other organic-molecule-based nanoparticles which show great promise for imaging-guided cancer precision therapy can be engineered through this method.

## Introduction

Although tremendous efforts have been made towards cancer theranostics for decades, it remains one of the leading causes of mortality worldwide 1-5. To date, phototherapy including photodynamic therapy (PDT) and photothermal therapy (PTT) has attracted considerable attention and emerged as a promising strategy in the personalized treatment of cancers due to their advantages such as minimized adverse side effect, locally selective treatment, minimal invasiveness, and negligible drug tolerance 6, 7. PDT always involves photosensitizers to produce reactive oxygen species (ROS) under light irradiation in the presence of oxygen, resulting in apoptosis and necrosis of tumor cells 8, 9. However, the efficacy of PDT has always been significantly affected by hypoxic microenvironments of the tumor tissues, limiting its extensive clinical applications 10-12. By contrast, PTT relies on the heat energy transferred from light energy with the aid of photothermal agents to damage the cancer cells, the efficacy of which has not been influenced with the hypoxic microenvironments of tumors 13-15. More interestingly, photoacoustic (PA) imaging, a noninvasive hybrid imaging approach with deep penetration and high sensitivity, can sense acoustic pressure waves generated from absorbed photon energy, which has involved the process of thermal expansion as PTT 16-18. Therefore, it is possible to monitor tumor location and evaluate the treatment efficiency by designing theranostic agents for PA imaging-guided PTT 19, 20.

Presently, theranostic agents with strong optical absorption in the near-infrared (NIR) biological window (700 - 1100 nm) have been extensively developed due to their deep tissue penetration and minimal photodamage to biological tissues 21 including inorganic 22-26 and organic nanomaterials 27-30. Compared to inorganic nanomaterials, organic nanoagents have exhibited numerous advantages such as excellent biocompatibility, tunable chemical structures, and potential biodegradability 31, 32. In particular, nanoparticles (NPs) based on BODIPY dyes that are a class of representative organic small molecules, have been widely developed for various cancer theranostics applications due to their excellent optoelectronic property and easy functionalization property 33-35. According to the structures of BODIPY dyes, they can be classified into two main categories including *meso*-carbon-BODIPY and Aza-BODIPY 36. Although *meso*-carbon-BODIPY has been increasingly explored for fluorescence bioimaging 37, 38, less extensive research of Aza-BODIPY is conducted due to the conspicuous success of the phthalocyanine dyes and laborious synthetic method of azadipyrromethene 39. Despite the fact that, Aza-BODIPY was synthesized 40,41 and used as the indicator 42, PA probe 43, 44, and photosensitizer 45. Further studies also showed that Aza-BODIPY is an attractive platform to reach NIR absorption because the highest occupied molecular orbital (HOMO)-lowest unoccupied molecular orbital (LUMO) gap is narrowed by replacing the *meso*-carbon with an Aza-nitrogen 46. Various elegant approaches have been presented to obtain Aza-BODIPY with NIR absorptions and emissions of including extension of the π-conjugation 47, 48, planarization of the π-system through reducing the torsion angles 49, and rigidification of rotatable moieties 50. It has attracted attentions to formulate a heptacyclic B, O-chelated constrained scaffold due to the noticeable bathochromic shift in the absorption and emission 51, 52. However, the application of this category of Aza-BODIPY is still in its infancy, which is mainly designed as electron donor for organic solar cells 53, 54. Therefore, it is of great significance to develop heptacyclic B, O-chelated Aza-BODIPY structure molecules and broaden their applications in biomedical fields, which might be an interesting add-on to the present organic nanoprobes for cancer theranostics.

**Scheme 1:**
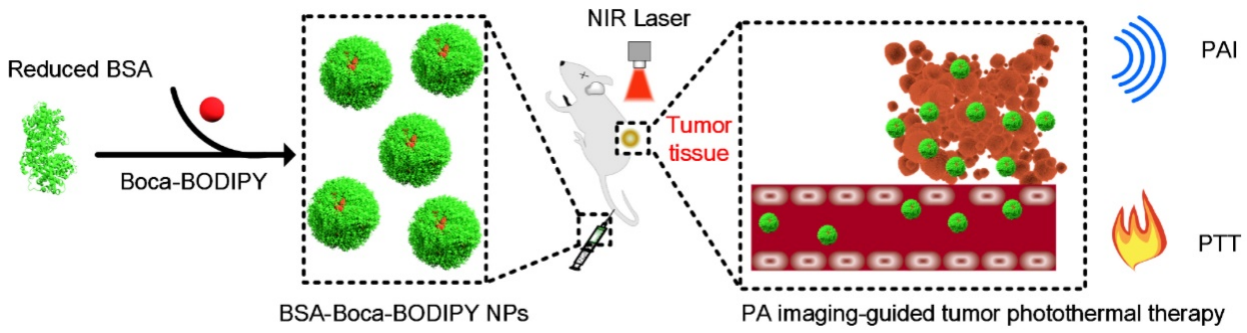
Schematic illustration of the construction of BSA-Boca-BODIPY NPs for in vivo photoacoustic imaging-guided photothermal treatment.

In this study, a novel heptacyclic B, O-chelated Aza-BODIPY (Boca-BODIPY) molecule with strong NIR absorption is designed through molecular engineering. By introducing the heavy atoms and alkyl chains, high photothermal conversion efficiency of the molecule can be achieved due to the heavy-atom effect and its interaction with protein is also promoted. The Boca-BODIPY molecule is further encapsulated in reduced bovine serum albumin (BSA) through self-assembly (BSA-Boca-BODIPY) to circumvent its poor aqueous solubility for biological applications. More importantly, the organic-molecule-based NPs have concurrently served as contrast agents for PA imaging and photothermal agents for PTT in vitro and in vivo at a low concentration (**Scheme 1**). The as-prepared BSA-Boca-BODIPY NPs possess several unique advantages. (1) Strong NIR absorption with negligible fluorescence and reactive oxygen species (ROS) generation, leading to enhanced PA imaging ability and high photothermal conversion efficiency; (2) good water-dispersible, excellent stability and biocompatibility ascribing to the addition of alkyl chains on the organic molecule that facilitate its interaction with BSA; (3) adequate hydrodynamic size, resulting in effective passive tumor targeting through enhanced permeability and retention (EPR) effect. This study represents the first demonstration of BSA-Boca-BODIPY NPs that can achieve highly contrast-enhanced PA imaging and imaging-guided high-efficiency cancer PTT. As a result, this study prospectively proposes a strategy to design organic-molecule-based NPs with unique advantages through molecular engineering for future personalized medicine.

## Experimental

### Chemicals

2,4-dihydroxyacetophenone, 1-bromooctane, potassium carbonate (K_2_CO_3_), potassium iodide (KI), magnesium sulfate (MgSO_4_), potassium hydroxide (KOH), 3-bromobenzaldehyde, glutathione (GSH), and 3-(4,5-dimethylthiazol-2-yl)-2,5-diphenyltetrazolium bromide (MTT) were all obtained from Sigma-Aldrich. Tetrahydrofuran (THF) was received from Tokyo Chemical Industry Co. Ltd. Dichloromethane (CH_2_Cl_2_), chloroform (CHCl_3_), and hexane were all purchased from Anaqua Global International Inc. Limited. SYTO 9 and propidium iodide (PI) were received from Thermal Fisher. Ethanol (EtOH) and methanol were obtained from Merk. Bovine serum albumin (BSA) was purchased from Aladdin. All commercial reagents were used as received unless otherwise stated. Ultrapure water (18.25 MΩ·cm, 25 °C) was used in the experiments.

### Characterization

^1^H and ^13^C NMR spectra were recorded on a Varian instrument (400 MHz and 100 MHz, respectively) and internally referenced to tetramethylsilane signal or residual proton solvent signals. Data for ^1^H NMR were recorded as follows: chemical shift (δ, ppm), multiplicity (s, singlet; d, doublet; t, triplet; q, quartet; m, multiplet), coupling constant (Hz), integration. Data for ^13^C NMR are reported in terms of chemical shift (δ, ppm). High resolution mass spectra for the compounds were done by an LTQ-Orbitrap instrument (ESI) (Thermo Fisher Scientific, USA). The optical absorption spectrum of the Boca-BODIPY and BSA-Boca-BODIPY NPs were measured by a Shimadzu UV-1800 UV-Vis absorption spectrophotometer. Transmission electron microscopy (TEM) image was conducted using an FEI Tecnai G20 transmission microscope at 200 kV. For the TEM observation, 10 µL of the BSA-Boca-BODIPY NPs with an adequate concentration was dropped on a carbon-coated copper grid and then dried under ambient environment. Hydrodynamic diameter and zeta potential of the BSA-Boca-BODIPY NPs were characterized using Malvern Nano-ZS Particle Sizer. Photoacoustic characterization was conducted with a home-made PA system.

### Synthesis of Boca-BODIPY

1-(2-hydroxy-4-(octyloxy)phenyl)ethan-1-one (1). A mixture of 2,4-dihydroxyacetophenone (5.00 g, 33.0 mmol), 1-bromooctane (6.34 g, 33.0 mmol) and K_2_CO_3_ (4.56 g, 33.0 mmol) was refluxed in acetone (250 ml) in presence of catalytic amount KI for 24 h. The reaction mixture was filtered and concentrated. To the solution was added an aqueous solution of KOH (0.2 M, 100 ml) and extracted with CH_2_Cl_2_ (3×100 ml). The organic layer was dried over MgSO_4_, filtered and the solvent was evaporated under reduced pressure. The crude product was chromatographed using CH_2_Cl_2_ as eluting solvent to give the title compound **1** as a colorless viscous liquid (7.14 g, 82%). ^1^H NMR (400 MHz, Chloroform-d) δ 12.76 (d, J = 1.4 Hz, 1H), 7.64 (dd, J = 8.9, 1.3 Hz, 1H), 6.49 - 6.39 (m, 2H), 4.01 (td, J = 6.5, 1.3 Hz, 2H), 2.57 (d, J = 1.4 Hz, 3H), 1.81 (pd, J = 6.7, 1.3 Hz, 2H), 1.46 (s, 2H), 1.36 - 1.30 (m, 8H), 0.91 (td, J = 5.5, 2.9 Hz, 3H). ^13^C NMR (101 MHz, CDCl_3_) δ 202.5, 165.8, 165.3, 132.2, 113.7, 108.0, 101.3, 68.4, 31.8, 29.3, 29.2, 29.0, 26.2, 25.9, 22.7, 14.1.

(E)-3-(3-bromophenyl)-1-(2-hydroxy-4-(octyloxy)phenyl)prop-2-en-1-one (3). 10.00 g 1-(2-hydroxy-4-(octyloxy)phenyl)ethan-1-one (**1**) (37.83 mmol) and 7.00 g 3-bromobenzaldehyde (**2**) (37.83 mmol) were dissolved in 500 mL EtOH, which was cooled to 0℃. And 22 mL 50% NaOH solution was added in batches of 1 mL while sustained the solution temperature under 0℃. The mixture was stirred overnight at room temperature. The mixture was poured into ice-water, and the pH of the mixture was adjusted to 4 using 5 M HCl. The resulting precipitate was filtered off and recrystallized from EtOH to afford 3a (4.00 g, 75%) as a yellow crystal. ^1^H NMR (400 MHz, Chloroform-d) δ 13.32 (s, 1H), 7.83 - 7.79 (m, 3H), 7.58 - 7.50 (m, 3H), 7.30 (t, J = 7.9 Hz, 1H), 6.51 - 6.44 (m, 2H), 4.01 (t, J = 6.5 Hz, 2H), 1.82 - 1.78 (m, 2H), 1.45 (q, J = 6.9 Hz, 2H), 1.38 - 1.24 (m, 8H), 0.93 - 0.85 (m, 3H). ^13^C NMR (101 MHz, CDCl_3_) δ 191.3, 166.8, 166.1, 142.4, 137.0, 133.3, 131.2, 130.8, 130.5, 127.4, 123.1, 121.8, 113.8, 108.3, 101.5, 68.5, 31.8, 29.3, 29.2, 29.0, 26.0, 22.7, 14.1.

(Z)-3-(3-bromophenyl)-1-(2-hydroxy-4-(octyloxy)phenyl)-4-nitrobut-2-en-1-one (4). The obtained compound **3** (8.00 g, 18.55 mmol) was dissolved in 100 mL pre-dry methanol. 5.22 mL nitromethane (5.66 g, 92.73 mmol, 5 equivalents) and 9.46 mL diethylamine (6.78 g, 92.73 mmol, 5 equivalents) were added under N_2_ atmosphere, which was heated under reflux for 12 h. The reaction solution was cooled to room temperature and acidified with 5 M HCl to pH 4, extracted with CH_2_Cl_2_. The collected organic phase was dried with anhydrous MgSO_4_ and concentrated. The residue was purified on silica gel (petroleum ether/ethyl acetate, v/v, 10 : 1) to afford 4a as a yellow oil (7.67 g, 84%). ^1^H NMR (400 MHz, Chloroform-d) δ 12.42 (s, 1H), 7.58 (d, J = 9.0 Hz, 1H), 7.48 - 7.39 (m, 2H), 7.27 - 7.17 (m, 2H), 6.48 - 6.37 (m, 2H), 4.82 (dd, J = 12.7, 6.4 Hz, 1H), 4.67 (dd, J = 12.8, 8.2 Hz, 1H), 4.26 - 4.17 (m, 1H), 3.99 (t, J = 6.6 Hz, 2H), 3.44 - 3.29 (m, 2H), 1.80 (dt, J = 14.7, 6.7 Hz, 2H), 1.52 - 1.41 (m, 1H), 1.46 (s, 1H), 1.35 - 1.28 (m, 8H), 0.95 - 0.90 (m, 3H). ^13^C NMR (101 MHz, CDCl_3_) δ 199.8, 166.2, 165.5, 141.2, 131.2, 131.0, 130.7, 130.5, 126.3, 123.1, 112.9, 108.6, 101.5, 79.2, 68.6, 40.4, 38.8, 31.8, 29.3, 29.2, 28.9, 25.9, 22.7, 14.1.

Boca-BODIPY. A solution of **4** (6.00 g, 12.18 mmol) and ammonium acetate (33.81 g, 438.66 mmol, 36.0 equivalents) in EtOH (120 mL) was heated under reflux for 72 h. After the compound **4** was consumed completely and cooled to room temperature, the reaction solution was concentrated and 150 mL CHCl_3_ was added into the residue. The organic solution was washed with water and filtered through Celite. The resulting residue was dissolved into toluene (180 mL). To the above solution was added Et_3_N (2.16 mL, 1.57 g, 1.27 equivalent) and BF_3_Et_2_O (3.12 mL, 3.51 g, 2.03 equivalents). After reaction under reflux overnight, the solvent was removed, and the residue was washed with saline and extracted with CHCl_3_. The solution was concentrated under reduced pressure to give crude product, which was purified by flash chromatograph (Hexane/ dichloromethane, v/v, 1 : 1) on silica gel to afford Boca-BODIPY as black solid in 30% overall yield (1.59 g). ^1^H NMR (400 MHz, Chloroform-d) δ 8.33 (t, J = 1.8 Hz, 2H), 8.16 - 8.09 (m, 2H), 7.70 (d, J = 8.8 Hz, 2H), 7.52 (ddd, J = 8.0, 2.0, 1.0 Hz, 2H), 7.38 (t, J = 7.9 Hz, 2H), 7.13 (s, 2H), 6.71 (dd, J = 8.7, 2.4 Hz, 2H), 6.55 (d, J = 2.4 Hz, 2H), 3.97 (qt, J = 9.3, 6.6 Hz, 4H), 1.85 - 1.76 (m, 4H), 1.45 (td, J = 10.7, 9.2, 6.1 Hz, 4H), 1.34 (td, J = 8.6, 3.8 Hz, 16H), 0.91 (d, J = 4.1 Hz, 6H). ^13^C NMR (101 MHz, CDCl_3_) δ 164.3, 158.0, 149.2, 144.3, 139.0, 134.5, 131.7, 131.4, 130.3, 127.6, 127.2, 122.8, 113.1, 112.1, 110.6, 104.2, 68.4, 31.8, 29.3, 29.2, 29.1, 26.0, 22.7, 14.1. HRMS-ESI calcd for C_48_H_50_BBr_2_N_3_O_4_ ([M+H]^+^) was 904.5700, found 904.2421.

**Boca-BODIPY without Br:** Boca-BODIPY molecule without Br atom was synthesized from the starting compound 1-(2-hydroxy-4-(octyloxy)phenyl)-4-nitro-3-phenylbutan-1-one (7) (1.59 g 3.83 mmol), ammonium acetate (27.75 g, 359.98 mmol) and 1-butanol (50 mL) were heated under reflux for 24 h. The reaction was cooled to room temperature and the solvent was removed under reduced pressure and the solid was re-dissolved in DCM and washed with water three times (3 × 50 mL). The organic layer was collected and concentrated. The residue was washed with ethanol and dried by vacuum oven at 40 ℃, it was dissolved in dry toluene (30 mL). Diisopropylethylamine (DIPEA, 3.19 g, 24.68 mmol) and boron trifluoride diethyl etherate (BF_3_·OEt_2_, 4.67 g, 32.91 mmol) were added and then the mixture was refluxed for 12 h under nitrogen. The solution was washed with water three times (3 × 50 mL) and dried over anhydrous sodium sulfate. The crude product was purified by column chromatography and recrystallized from a hexane-THF mixture. ^1^H NMR (400 MHz, Chloroform-*d*) δ 8.19 - 8.11 (m, 4H), 7.70 (d, *J* = 8.8 Hz, 2H), 7.54 - 7.37 (m, 6H), 7.12 (s, 2H), 6.69 (dd, *J* = 8.8, 2.4 Hz, 2H), 6.56 (d, *J* = 2.4 Hz, 2H), 3.95 (qt, *J* = 9.3, 6.7 Hz, 4H), 1.83 - 1.71 (m, 4H), 1.50 - 1.26 (m, 20H), 0.97 - 0.83 (m, 6H). ^13^C NMR (101 MHz, CDCl_3_) δ 162.92, 156.95, 147.96, 143.35, 140.04, 131.61, 127.86, 127.82, 127.57, 126.48, 111.50, 111.20, 109.27, 103.21, 67.31, 30.76, 28.67, 28.30, 28.24, 28.18, 28.03, 24.94, 21.61, 13.06. HRMS-ESI calcd for C_48_H_52_BN_3_O_4_ ([M+H]^+^) was 746.7780, found 746.4273.

### Preparation of BSA-Boca-BODIPY NPs

To prepare the BSA-Boca-BODIPYNPs, 40 mg mL^-1^ BSA was first dissolved in double-distilled water in the presence with 30 mg mL^-1^ glutathione (GSH) at 37℃ for 30 min. And then 1 mg mL^-1^ Boca-BODIPY molecules dissolved in tetrahydrofuran (THF) was added into the solution slowly under vigorous stirring for 2 h. After the reaction, the mixture was centrifuged to remove free Boca-BODIPY molecules, GSH, and the THF by ultrafiltration tube (cutoff MW:10 KD). At last, the BSA-Boca-BODIPY NPs were obtained and stored at 4℃ for further application.

### Photothermal ability of the BSA-Boca-BODIPY NPs

To assess the photothermal ability of the BSA-Boca-BODIPY NPs, the temperature of NPs with different concentrations ranging from 0 to 50 μg mL^-1^ was monitored using the infrared thermal imaging camera (Ti400, Fluke, USA) at an interval of 10 s under the irradiation of 808 nm laser (Beijing Laserwave OptoElectronics Technology Co., Ltd) at the power density at 0.75 w cm^-2^. The NPs were also exposed to different power intensity of the 808 nm laser including 0.25 w cm^-2^, 0.5 w cm^-2^, 0.75 w cm^-2^ at the concentration of 50 μg mL^-1^, the temperature of the solution was then recorded to evaluate the effect of the power density. The photothermal conversion efficiency (η) of the BSA-Boca-BODIPY NPs was further calculated according to the Equation (1) previously reported as described follows:


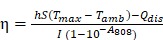
 (1)

where *h* is representative heat transfer coefficient, *S* represents the surface area of the container, *T_max_* and* T_amb_* indicate the maximum equilibrium temperature and ambient temperature of the system under the laser irradiation respectively, Q_dis_ is the heat energy dissipation from the light absorbed by the solvent, *I* expresses the power density of the laser used in the experiment, and *A* is the absorbance of the BSA-BODIY NPs at 808 nm.

The value of *hS* can be obtained using the Equation (2):


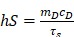
 (2)

Where τ_s_ is the BSA-Boca-BODIPY NPs system time constant, m_D_ indicates the mass of the solvent, and C_D_ is the heat capacity of the double-distilled water.

τ_s_ can be measured by calculating the data of the temperature variation ratio after turn of the laser irradiation vs negative natural logarithm of driving force temperature.


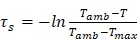
 (3)

According to the data, τ_s_ was determined to be 159 s. And then all the data can be acquired, the photothermal conversion efficiency (*η*) of BSA-Boca-BODIPY NPs can be calculated.

hS = m_D_ C_D_ /τ_s_ = 0.0132; Q_dis_ = 0.0352; T_max_ - T_amb_ = 27.5; I = 0.75; A_808_ = 0.592

η = (0.0132 x 27.5-0.0352) / (0.75 x (1-10^-0.592^)) = 58.7%

### ROS generation ability of the BSA-Boca-BODIPY NPs

2′,7′-dichlorofluorescin diacetate, a highly sensitive ROS indicator, was used to monitor the ROS generation. The Ce6 was used as a positive control in the experiment. Generation of ROS of three groups including ROS sensor, BSA-Boca-BODIPY NPs plus ROS sensor, and positive control in the presence of ROS indicator were measured by detecting enhanced fluorescence of ROS sensor (λ _excitation_ = 504 nm).

### In vitro PA imaging

The PA signals originated from the as-prepared BSA-Boca-BODIPY NPs were measured by our home-made PA system. To build the system, the nanosecond pulsed OPO laser (SureliteI-20, Continuum, USA) was adopted as the energy source to excite the materials, and the ultrasound transducer of 1MHz central frequency with bandwidth ranging from 0.65 to1.18 MHz (V303-SU, Olympus NDT) was used to detect the generated acoustic signal. Furthermore, the data acquisition module (Tektronix TDS3054 Digital Phosphor Oscilloscope) and motorized 3D scanning stage (PSA2000-11, Zolix, Beijing, China) were also used to construct the system. The PA spectra of BSA-Boca-BODIPY NPs was obtained by recording the PA signals of agarose gel phantom containing 10 μg mL^-1^ BSA-Boca-BODIPY NPs with the interval of 10 nm ranging from 680 nm to 950 nm. The PA intensities were measured six times at each wavelength. The PA stability of the BSA-Boca-BODIPY NPs was evaluated by monitoring the variation of PA intensity under the excitation of 800 nm laser at the energy density of about 6 mJ cm^-2^ for 5 x 10^4^ circles. The PA images and intensities were obtained by measuring the agarose gel phantoms with different concentrations of BSA-Boca-BODIPY NPs ranging from 0.625 μg mL^-1^ to 10 μg mL^-1^ under the same conditions. It should point out that water was used as coupling agent for all the PA experiments.

### Cell culture

For in vitro cell experiment, the 293T cell line and the 4T1 breast cancer cell line were cultured in Dulbecco's Modified Eagle Medium (DMEM, Gibco), which was supplemented with 10% (v/v) fetal bovine serum (FBS) and 1% (v/v) penicillin-streptomycin in a humidified incubator at 37 °C with 5% CO_2_.

### Cell uptake of the BSA-Boca-BODIPY NPs

To evaluate the cellular uptake of the BSA-Boca-BODIPY NPs, indocyanine green (ICG) with near-infrared fluorescence emission terminated by carboxyl group was conjugated on the surface of the NPs. Briefly, the carboxyl group of the ICG molecule dissolved in dry DMSO was first activated by EDC/NHS, which was dropped into the BSA-Boca-BODIPY NPs aqueous solution later. And then, the free ICG and DMSO were removed by ultrafiltration centrifugation (30 kD). All the procedures were carried out in dark. The fluorescence spectrum was further obtained to confirm the conjugation of the ICG.

After cultured in the confocal dish for 24 h, 4T1 cells were incubated with BSA-Boca-BODIPY-ICG for 12 h. Then, the cells were washed with PBS for three times. Subsequently, the cells were fixed with 4% paraformaldehyde solution for 10 min and 4′,6-diamidino-2-phenylindole (DAPI) for 3 min. Fluorescence images of the stained cells were further obtained by a confocal laser scanning microscope (CLSM, Leica TCS SP5, Germany), using 405 and 633 nm laser wavelength for DAPI and ICG excitation, respectively.

### In vitro cytotoxicity

The cytotoxicity of the as-obtained BSA-Boca-BODIPY NPs was investigated by measuring cell viability of 293T cells and the 4T1 breast cancer cells using MTT assay. 293T cells and 4T1 cells (5 × 10^3^ cells/per well, 100 µL) were seeded on 96-well plates for 12 h according to the above procedure, respectively. And then, the medium containing different concentrations of the BSA-Boca-BODIPY NPs (0, 3.125, 6.25, 12.5, 25, 50 µg/mL) was added into the wells respectively after removed the originated medium. After 24 h incubation at 37 °C, the cell viabilities were measured by the MTT assay. The cells incubated without BSA-Boca-BODIPY NPs was used as the blank control. Further, cell cytotoxicity of four groups 4T1 breast cancer cells including PBS-treated, NPs-treated, Laser-treated, NPs plus laser-treated were also measured using the MTT assay.

### SYTO 9/PI cell staining

The cells were first incubated with medium containing PBS or BSA-Boca-BODIPY NPs in 35 mm dishes for confocal imaging. After 12 h incubation, the laser treatment was conducted after replacement of the medium with DMEM (808 nm, 0.75 w cm^-2^). And then the cell was incubated for another 4 h before staining with the mixture of SYTO 9/PI. Fifteen minutes later, the dishes were washed with PBS three times. The fluorescence images of the samples under different treatment were recorded using confocal microscopy.

### Animals and tumor model

The experiments involved in animals were conducted by the approval of Animal Care and Usage Committee of University of Macau and Shenzhen Institutes of Advanced Technology, Chinese Academic of Sciences. Six-eight weeks BALB/c mice were selected to establish tumor model for in vivo PA imaging and PTT. The tumor cells were cultivated according to the procedure mentioned above. The subcutaneous tumor models were developed by injecting 2 × 10^6^ cells in PBS subcutaneously at the legs of the mice. The in vivo experiments were conducted when the tumor size reached to 100 mm^3^.

### In vivo PA imaging

The 4T1 tumor-bearing mice were intravenously injected with as-prepared BSA-Boca-BODIPY NPs at a dose of 0.5 mg kg^-1^ (body weight). The PA imaging were further conducted using the home-made PA system as described previously. The PA signals were obtained at different time intervals (2 h, 5 h, 10 h, 12 h ,24 h) under the 800 nm excitation at the energy density of 6 mJ cm^-2^. It should be noted that the time was denoted as 0 h before injection of the contrast agent.

### In vivo photothermal therapy and histological analysis

For in vivo photothermal therapy, twenty 4T1 tumor-bearing mice were divided into four groups randomly (n = 5). Then BSA-Boca-BODIPY NPs or PBS were injected intravenously into the mice according to the different treatment as following: (1) PBS-treated; (2) BSA-Boca-BODIPY NPs-treated; (3) Laser-treated; and (4) BSA-Boca-BODIPY NPs plus laser-treated (laser wavelength: 808 nm; laser density: 0.75 w cm^-2^; irradiation time: 5 min). Tumor volumes and body weights of the tumor-bearing mice under different treatment were monitored. The mice were finally sacrificed to collect the tumor for H&E staining after 30 days. The major organs including heart, liver, spleen, lung, and kidney of normal mice were also collected after intravenously injected BSA-Boca-BODIPY NPs for a week to test the in vivo biocompatibility through H&E staining.

## Results and Discussion

### Synthesis and Characterization of Boca-BODIPY Molecules

**Scheme 2:**
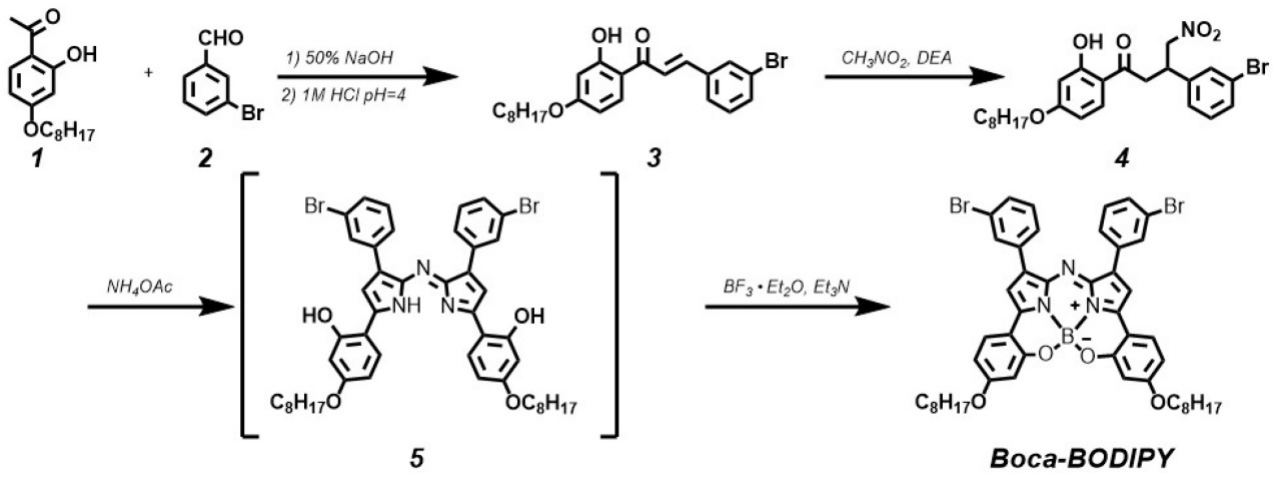
Synthetic route to compound Boca-BODIPY molecule.

**Figure 1:**
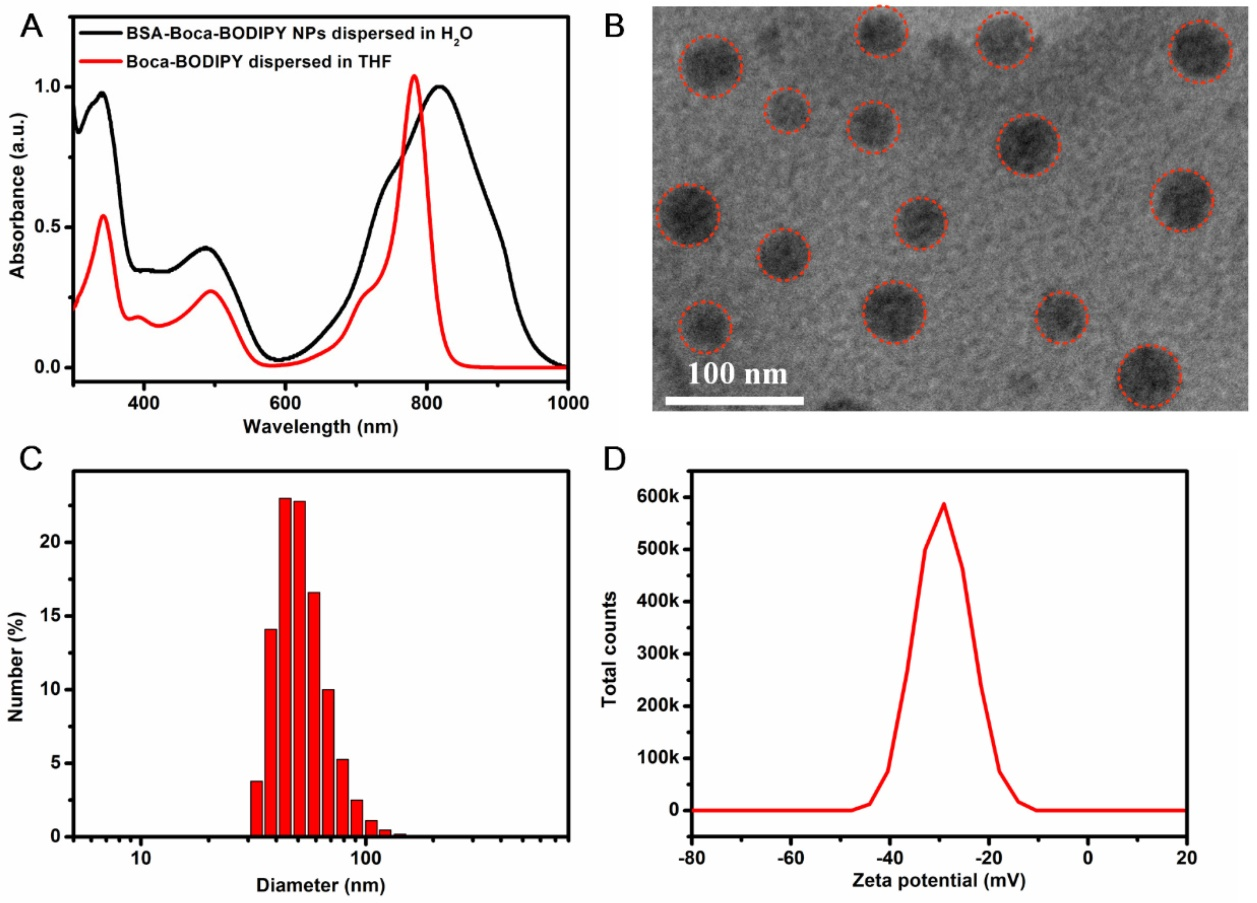
Characterization of the as-prepared BSA-Boca-BODIPY NPs. A) Uv-vis-NIR absorption spectra of the Boca-BODIPY dissolved in THF and the BSA-Boca-BODIPY NPs dispersed in water; B) Transmission Electron Micrograph (TEM) image of BSA-Boca-BODIPY NPs with an average size of 32 ± 3 nm, red circles were representative of the BSA-Boca-BODIPY NPs; C) Hydrodynamic diameter and D) zeta potential of the as-prepared BSA-Boca-BODIPY NPs dispersed in PBS measured by dynamic light scattering (DLS).

It is a creative approach to design BODIPY materials with NIR absorption. Interestingly, introducing heavy-atom substituents on the adequate position of aryl rings can give rise to nonradiative internal back-conversion to the ground state and inhibit the photosensitizer triplet to ground-state oxygen energy transfer, which is termed as “intramolecular external heavy atom (IEHA) effect”.^38^ In this study, to exploit Boca-BODIPY with NIR absorption, high PA signals, and photothermal conversion efficiency, bromine atoms, leading to IEHA effect, were introduced on the 1 and 7 positional aryl rings of heptacyclic B, O-chelated constrained scaffold. As PTT and PDT are competing pathways, the IEHA effect can facilitate the PA and PTT effect but inhibit the PDT effect,^38^ resulting in a more efficient theranostic agent. Another consideration for designing Boca-BODIPY is how to enhance the interaction between the dye and the carrier. Very recently, a single high-affinity fatty acid binding site is identified in serum albumin, which is different from Sudlow-sites I and II 55. Meanwhile, it is reported that indocyanine green (ICG) with N-alkyl side chains had strong interaction with HSA 56. Inspired by these two works, 2,4-dihydroxyacetophenone was alkylated with 1-bromooctane using KI as catalyst 57. Routine access to Boca-BODIPY included four reaction steps from 3-bromobenzaldehyde (2) and 1-(2-hydroxy-4-(octyloxy)phenyl)ethan-1-one (1). Addition of nitromethane to the α, β-unsaturated ketone 3 in presence of DEA produced 4 in 84% yield. Thereafter, the phenolic-substituted azadipyrromethene 5 was produced by refluxing compound 4 with ammonium acetate in EtOH for 72 h. Eventually, the unseparated 5 were converted into final product Boca-BODIPY through a one-pot chelation and intramolecular phenolic oxygen-fluorine displacement cascade reaction (**Scheme 2**). All the as-synthesized compounds were characterized by a Varian instrument. The ^1^HNMR, ^13^C NMR, and EMI-MS spectra were displayed in the supporting information (**Figure S**1- S10). The Boca-BODIPY molecule dissolved well in organic solvents. The absorption spectrum of the molecule dissolved in tetrahydrofuran (THF) was shown in **Figure 1**A, which exhibited three absorption peaks including 343 nm, 495 nm, and 782 nm. Additionally, the molar extinction coefficient at 782 nm of the as-synthesized Boca-BODIPY molecule is further determined to be about 8.5 x 10^4^ L mol^-1^ cm^-1^. To confirm the mentioned IEHA effect, Boca-BODIPY molecule without Br atom was synthesized as a control group (**Scheme S1**). NMR and ESI-MS were utilized to characterize the as-prepared Boca-BODIPY molecule without Br atom (**Figure S11- S14**). Further, we compared the ROS generation ability of the Boca-BODIPY molecules with and without Br atoms by measuring the fluorescence intensity of the singlet oxygen sensor green (SOSG) under the irradiation of NIR laser at the same condition. The fluorescence intensity at different time intervals were measured. As displayed in **Figure S15A**, the fluorescence intensity of the ROS indicator only increased a little under the NIR laser irradiation, indicating weak ROS generation ability of the Boca-BODIPY with Br atoms. In contrast, the Boca-BODIPY without Br atom group exhibited its huge capacity in producing ROS, resulting in dramatically increment of fluorescence intensity of SOSG (**Figure S15B**). The ^1^O_2_ quantitative analysis of the two kinds of molecules was performed using commercial indocyanine green (ICG) as a contrast. The slope of ICG was more than twice higher than that of Boca-BODIPY molecule with Br atoms, whereas, the slope of Boca-BODIPY molecule without Br atom is more than four times higher than that of ICG (**Figure S16**). It indicated poorer ROS generation ability of the Boca-BODIPY molecules with Br atoms than that of without Br atom. The interactions between Boca-BODIPY and BSA was determined by isothermal titration calorimetry (ITC) analysis. It indicated that the engineered Boca-BODIPY molecule can interact well with BSA** (Figure S17)**.

### Preparation and Characterization of BSA-Boca-BODIPY NPs

To explore the Boca-BODIPY as a candidate for biological applications, bovine serum albumin, a plasma protein containing abundant active groups was introduced to modify the organic molecules through self-assembly method in the presence of glutathione (GSH). The Boca-BODIPY molecules formed NPs in the assistance of the biocompatible protein, denoted as BSA-Boca-BODIPY NPs, which dispersed well in the aqueous solution. The absorption spectra of the organic Boca-BODIPY molecules and BSA-Boca-BODIPY NPs were recorded. As shown in Figure 1a, the BSA-Boca-BODIPY NPs exhibited strong absorption covering the NIR region of 700 - 950 nm with a characteristic peak at 817 nm, which was red-shifted about 30 nm compared with the Boca-BODIPY molecules dissolved in THF solution. It can be ascribed to the high order aggregation during the self- assembly formation of NPs which may increase the interactions of the molecules 58. The morphology and size of the BSA-Boca-BODIPY NPs were then measured by transmission electron microscopy (TEM). As demonstrated in Figure 1B, the as-prepared BSA-Boca-BODIPY NPs showed a uniform spherical structure with an average diameter of about 32 nm. It can also be observed that the NPs possessed good monodispersity in the TEM image, suggesting the BSA-Boca-BODIPY NPs dispersed well in aqueous solution. In addition, hydrodynamic diameter and zeta potential of the BSA-Boca-BODIPY NPs dispersed in PBS were characterized using dynamic light scattering (DLS). The hydrophilic diameter was reached to 53.56 nm, which was larger than that displayed in the TEM image (Figure 1C). The reason for the difference of the size measured by different instruments was that TEM exhibited the diameter dried on the copper grid, while the size measured by DLS represented the diameter in aqueous solution. Furthermore, the adequate size of the BSA-Boca-BODIPY NPs was facilitated their tumor-preferential accumulation by the enhanced permeability and retention effect (EPR effect). As demonstrated in Figure 1D, the zeta potential of the BSA-Boca-BODIPY NPs was 30.1 mV below zero, which was suitable for protecting the NPs from aggregation. We also monitored the time-dependent leaching of the as-prepared BSA-Boca-BODIPY nanoparticles by measuring its absorbance variation. As shown in the** Figure S18**, the absorption intensity of the BSA-Boca-BODIPY nanoparticles decreased less than 4% at first 6 h and decreased 6% at 12 h. The absorption intensity remained more than 90% at 72 h, indicating only a little Boca-BODIPY molecules was released from the protein-based NPs. Therefore, the as-prepared BSA-Boca-BODIPY NPs could be an excellent candidate for biological applications due to its strong NIR absorption, adequate particle size, and negative zeta potential.

### In Vitro PA imaging Ability of the BSA-Boca-BODIPY NPs

**Figure 2:**
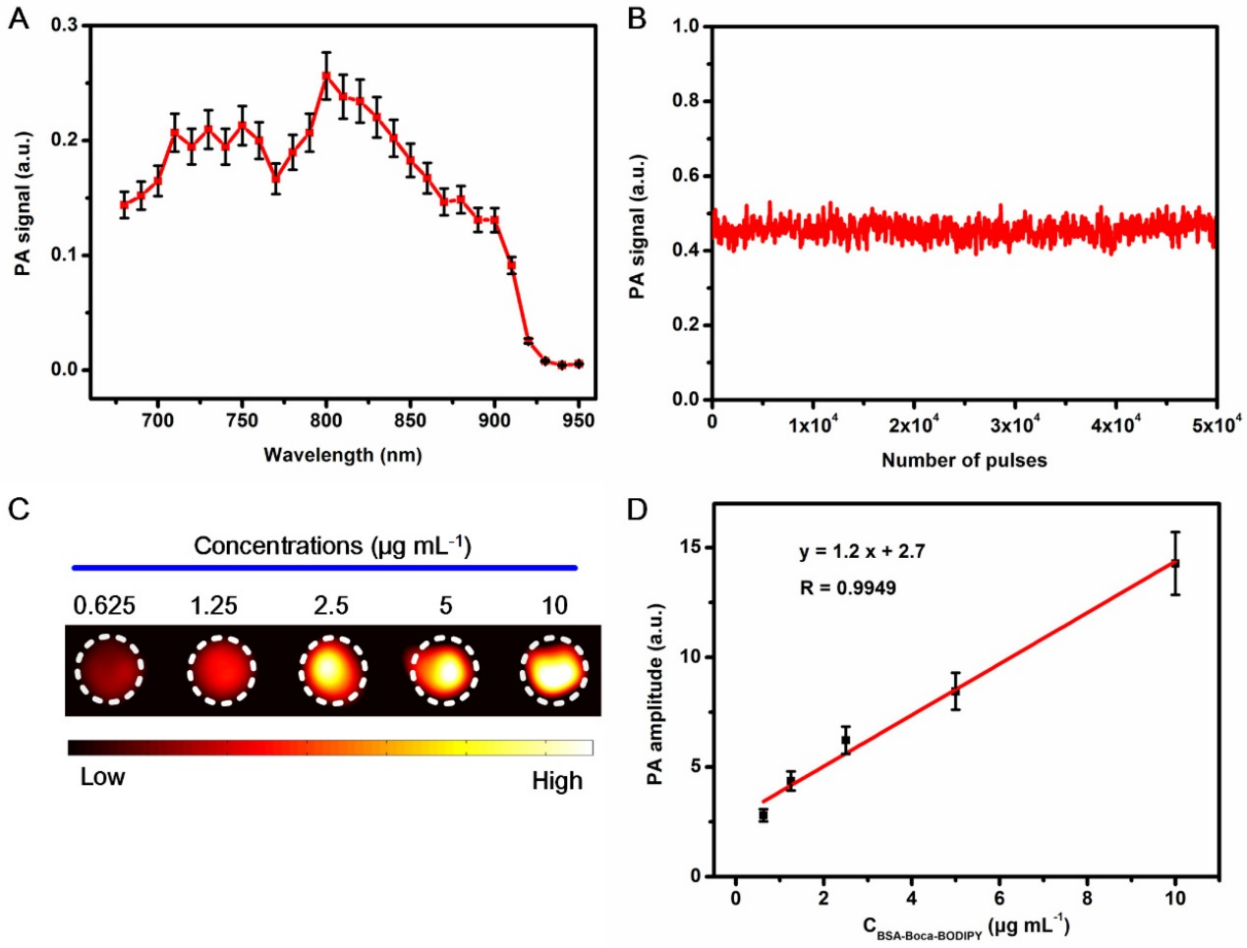
Measurement of in vitro PA imaging abilities of the BSA-Boca-BODIPY NPs. A) PA spectrum of BSA-Boca-BODIPY NPs obtained in agar phantom;B) PA signals of BSA-Boca-BODIPY NPs in agar phantom versus number of laser pulses; C) PA images of BSA-Boca-BODIPY NPs under excitation at 800 nm at different concentrations including 0.625, 1.25, 2.5, 5, 10 μg mL^-1^; D) The PA amplitudes at 800 nm as a function of concentrations of BSA-Boca-BODIPY NPs.

As the developed BSA-Boca-BODIPY NPs had strong absorption in the NIR region and nearly no fluorescence emission (**Figure S19**), it deduced to generate PA signals under laser excitation. The PA signals of BSA-Boca-BODIPY NPs at the wavelengths ranging from 680 nm to 950 nm were recorded by the home-made PA system reported previously. The strong PA signals can be detected from 680 nm to 900 nm with a characteristic peak at 800 nm which was nearly over the NIR biological window (**Figure 2**A). It can be observed that the tendency of the PA spectrum of the BSA-Boca-BODIPY NPs was nearly identical with the absorption spectrum. However, the characteristic peak of the PA spectrum was blue-shifted about 20 nm compared with the absorption spectrum. The deviation between the PA signals and absorption spectrum may be ascribed to the different optical illumination parameters and photophysical processes of the two kinds of spectra. This phenomenon was also observed in other PA agents 59. PA signals of the BSA-Boca-BODIPY NPs under the laser irradiation was recorded to assess the photostability. As shown in Figure 2B, the PA signals were nearly no change after exposure to the laser with 6 mJ cm^-2^ energy density at the wavelength of 800 nm for 5.0 × 10^4^ pulses. It suggested that the BSA-Boca-BODIPY NPs possessed excellent photostability, indicating its feasibility for possible long-time PA imaging. After obtained the characteristic peak and good stability under the laser excitation, PA images of the BSA-Boca-BODIPY NPs under 800 nm laser excitation at different concentrations ranging from 0.625 μg mL^-1^ to 10 μg mL^-1^ were further obtained. As can be observed in Figure 2C, the BSA-Boca-BODIPY NPs can generate PA signals at a low concentration. Moreover, the PA intensities were increased with the concentration increasing, indicating the PA signals originated from the BODIPY molecules. It was also found a good linear relationship (R = 0.9949) between the PA signals and the concentrations of BSA-Boca-BODIPY NPs, suggesting it possible for signal quantification. Therefore, the results demonstrated the BSA-Boca-BODIPY NPs were excellent agents for PA imaging.

### Photothermal Property and Stability of the BSA-Boca-BODIPY NPs

**Figure 3:**
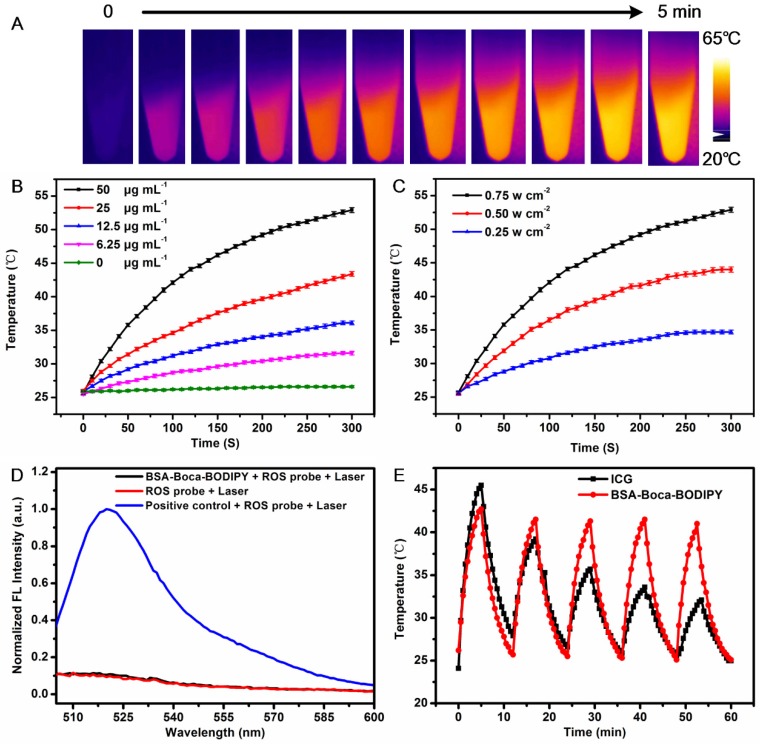
Photothermal properties of BSA-Boca-BODIPY NPs. A) Photothermal images of BSA-Boca-BODIPY NPs solution (50 μg mL^-1^) under the irradiation of 808 nm laser at the power density of 0.75 w cm^-2^; B) The temperature variation of the solution containing different concentrations of BSA-Boca-BODIPY NPs (0, 6.25, 12.5, 25, 50 μg mL^-1^) under the irradiation of 808 nm laser at the power density of 0.75 w cm^-2^; C) The temperature curves of BSA-Boca-BODIPY NPs at the concentration of 50 μg mL^-1^ under the irradiation of 808 nm laser at different power densities; D) Fluorescence of ROS probe in the presence/absence of the BSA-Boca-BODIPY NPs and positive control group under laser irradiation; E) Temperature variations of the BSA-Boca-BODIPY NPs and ICG under 808 nm laser irradiation at a power density of 0.75 w cm^-2^ for five light on/off cycles.

The photothermal effect of the as-prepared BSA-Boca-BODIPY NPs was further investigated. As shown in **Figure 3**A, the thermal images of the BSA-Boca-BODIPY NPs solutions under the irradiation of continuous 808 nm laser for 5 min were recorded using an infrared thermal imaging camera. It observed the temperature was increased with the irradiation time, indicating the BSA-Boca-BODIPY NPs can transfer light energy into heat energy. We further monitored the photothermal effect of the BSA-Boca-BODIPY NPs at different concentrations ranging from 0 to 50 μg mL^-1^. As displayed in Figure 3B, the temperature was increased quickly in the first 3 min, while the rate was decreased and reached a plateau within 5 min. It was also found that the temperature increment was dependent on the concentration of the BSA-Boca-BODIPY NPs. The relationship between the temperature and the power density of the laser was also studied. The temperature variation was up to about 30℃ at the concentration of 50 μg mL^-1^ under the irradiation of 0.75 w cm^-2^ 808 nm laser, while it was only increased 5℃ with the laser power density of 0.25 w cm^-2^. It demonstrated the temperature increment was also related to the laser power density (Figure 3C). Further, the photothermal conversion efficiency of the BSA-Boca-BODIPY NPs was assessed. The temperature of the BSA-Boca-BODIPY NPs solution was monitored as a function of the irradiation time of the 808 nm laser at 0.75 w cm^-2^. The laser was shut off immediately at the temperature reached a steady state, while the temperature was recorded continually (**Figure S20**). The photothermal conversion efficiency was then calculated according to the formula described in the experimental part. It was estimated to be about 58.7%, which was much higher than that of reported indocyanine green (ICG) NPs (17.3%) and commercial Au nanorods (21%) 60, 61. We had also compared the photothermal conversion efficiency with other reported inorganic and organic nanomaterials as listed in **Table 1**.

To investigate the possible reason for the high photothermal conversion efficiency, the reactive oxygen species (ROS) generation ability of the BSA-Boca-BODIPY NPs was measured using ROS probe. As displayed in Figure 3D, the fluorescence intensity of the ROS probe under the laser irradiation was nearly the same with that in the presence of the BSA-Boca-BODIPY NPs. While the fluorescence intensity was dramatically increased in the presence of positive control group. Thus, negligible ROS was generated by the BSA-Boca-BODIPY NPs which may be partly responsible for the high photothermal conversion efficiency 67. Therefore, it can be concluded that the as-prepared BSA-Boca-BODIPY NPs is an excellent candidate as a light-mediated thermal agent for cancer photothermal therapy.

Stability of the nanostructures have played important roles in the biological applications since the property variations of NPs may mislead the diagnosis and therapy of diseases. It may also introduce uncontrollable toxicity to the living systems 19, 68. Therefore, it is essential to evaluate the stability of the as-prepared BSA-Boca-BODIPY NPs before used in biological fields. As displayed in Figure 3E, we found that the temperature of the as-obtained BSA-Boca-BODIPY NPs maintained the same even after five irradiation cycles under the irradiation of 808 nm laser. In contrast, the temperature of the ICG, an FDA-approved commercially available dye, was not able to last under the same irradiation condition. It indicated the BSA-Boca-BODIPY NPs had excellent photothermal stability. The photographs of the BSA-Boca-BODIPY NPs dispersed in water were recorded to measure its storage stability. As shown in **Figure S**21A, there was no aggregation can be found after storage in the ambient environment for half a month and the signals exhibited a negliable decrease (**Figure S**22), suggesting its excellent colloid stability. The pH stability of the BSA-Boca-BODIPY NPs was further observed by dispersing the BSA-Boca-BODIPY NPs in buffer with different pH values ranging from 4.5 to 10.5. It observed that the BSA-Boca-BODIPY NPs kept their transparent deep yellow in the different pH values (Figure S21B). The results indicated that the BSA-Boca-BODIPY NPs stayed stable in the broad range of pH values. It can ascribe to protection effect of the protein shell. As the NPs were designed as theragnostic agents for tumor therapy, the stability of the BSA-Boca-BODIPY NPs in H_2_O_2_ which can be generated in the tumor microenvironment was further measured. The photograph demonstrated that no precipitation or color change of the BSA-Boca-BODIPY NPs can be found even at the concentration up to 100 mM (Figure S21C). The results exhibited the BSA-Boca-BODIPY NPs possessed good stabilities for biological applications.

### In Vitro Cytotoxicity and Photothermal Effect of the BSA-Boca-BODIPY NPs

**Figure 4:**
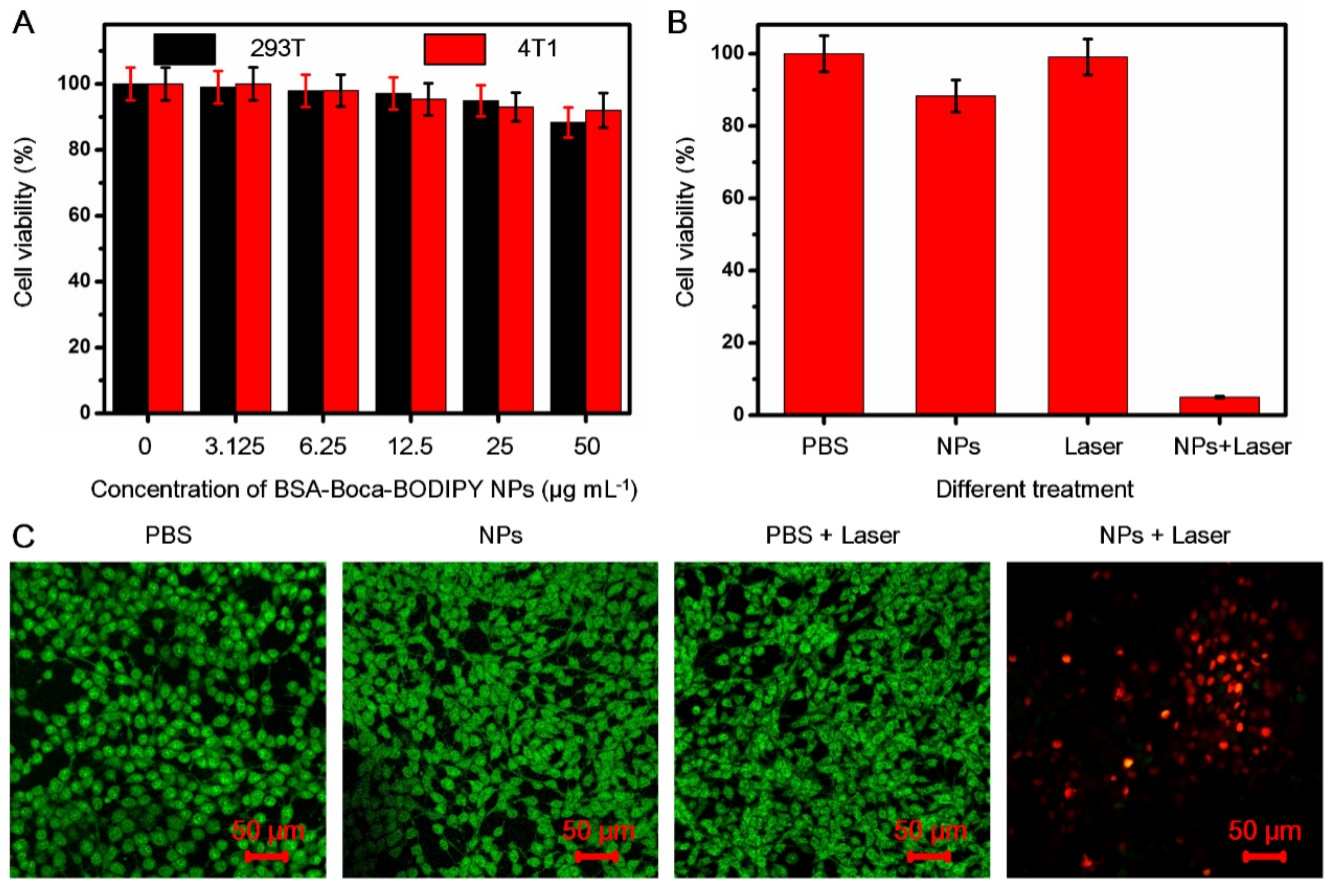
In vitro cytotoxicity of the BSA-Boca-BODIPY NPs with/without laser irradiation. A) Cell viabilities of 293T cells and 4T1 breast cancer cells incubated with the as-prepared BSA-Boca-BODIPY NPs at various concentrations ranging from 0 μg mL^-1^ to 50 μg mL^-1^; B) Cell viabilities of 4T1 breast cancer cells with different treatments including PBS, NPs (50 μg mL^-1^), laser (808 nm, 0.75 w cm^-2^, 5 min), and NPs plus laser (50 μg mL^-1^, 808 nm, 0.75 w cm^-2^, 5 min); C) Confocal fluorescence images of 4T1 breast cancer cells costained with SYTO 9 (green, live cells) and PI (red, dead cells) after different treatments including PBS, BSA-Boca-BODIPY NPs, laser, and BSA-Boca-BODIPY NPs under laser irradiation.

For the following potential in vivo biological applications, the biocompatibility of the as-prepared BSA-Boca-BODIPY NPs was first investigated. The cytotoxicity was evaluated by testing the cell viability of human embryonic kidney 293T cell line and the 4T1 mammary carcinoma cell line cultured in different concentrations of BSA-Boca-BODIPY NPs ranging from 0 μg mL^-1^ (control) to 50 μg mL^-1^ using standard MTT assay at 24 h. The cell viabilities of both 293T cells and 4T1 breast cancer cells were nearly no change in the broad concentration range of BSA-Boca-BODIPY NPs, even at a concentration up to 50 μg mL^-1^, indicating that the BSA-Boca-BODIPY NPs possessed good biocompatibility at this dosage (**Figure 4**A). The extent of apoptosis was also measured by a flow cytometer. As shown in **Figure S23** , there was nearly no difference between the BSA-Boca-BODIPY NPs group and PBS group. It demonstrated that the BSA-Boca-BODIPY NPs at the concentration of 50 μg mL^-1^ had no additional adverse effect on 4T1 cells compared to that of the PBS control. To evaluate the cellular uptake of the BSA-Boca-BODIPY NPs, indocyanine green (ICG) with NIR fluorescence emission terminated by carboxyl group was conjugated on the surface of the NPs. As shown in **Figure S24**, strong NIR fluorescence of the ICG-conjugated BSA-Boca-BODIPY can be detected after the purification, indicating ICG was successfully linked on the BSA-Boca-BODIPY NPs. Fluorescence signal of ICG can be observed in the 4T1 cells after incubated with ICG-conjugated BSA-Boca-BODIPY NPs, demonstrating the as-obtained NPs can be internalized in the tumor cells (**Figure S25**). Further, the cell viabilities of the 4T1 breast cancer cells were measured after treatment with PBS alone, NPs alone, Laser alone and NPs plus Laser. As shown in Figure 4B and (**Figure S26A**), the cell viability of the group treated with NPs plus laser was dramatically decreased while no obvious change of the viabilities was observed in the other three groups. Besides the concentrations of the as-prepared BSA-Boca-BODIPY NPs, the power density of the laser also had great effect on the cell viability. The cell viability of the 4T1 breast cancer cells was over 80% at the power density of 0.25 w cm^-2^ while it dramatically decreased to about 5% at the power density of 0.75 w cm^-2^ (**Figure S26B**). It suggested that the cancer cells can be significantly damaged after incubation with BSA-Boca-BODIPY NPs under the 808 nm laser irradiation. Moreover, we had introduced SYTO 9 (stains live cells with green fluorescence)/PI (stains dead cells with red fluorescence), a live/dead cell double-staining kit, to visualize the viable and dead cancer cells by confocal fluorescence microscope after different treatments (Figure 4C). PBS-treated, NPs-treated, PBS plus Laser-treated groups exhibited strong green fluorescence, demonstrating no therapeutic effect of these treatments. In contrast, red fluorescence was observed in the NPs plus Laser-treated group, indicating photothermal effect of the as-prepared BSA-Boca-BODIPY NPs under laser irradiation. The results demonstrated the excellent biocompatibility and photothermal ability of the BSA-Boca-BODIPY NPs for destroying cancer cells in vitro.

### In Vivo PA and Fluorescence Imaging

**Figure 5:**
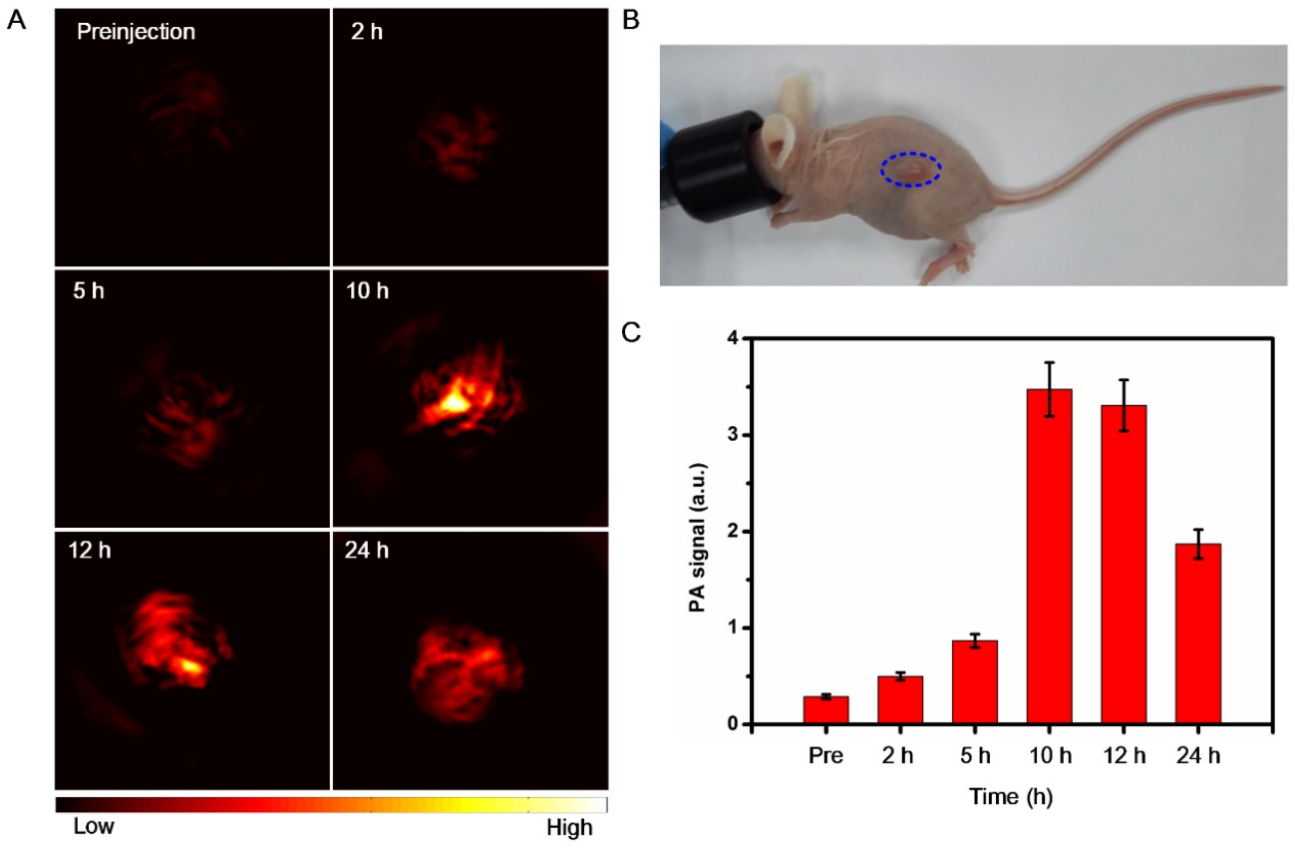
In vivo PA imaging of BSA-Boca-BODIPY NPs. (a) PA images before (0 h) and after tail-vein intravenous injection of BSA-Boca-BODIPY NPs at different time points (2 h, 5 h, 10 h, 12 h, 24 h) of 4T1 tumor-bearing nude mice; (b) Photograph of the 4T1 tumor-bearing nude mice, blue circle indicates the tumor; (c) Quantitative analysis of PA signals of the tumor at different time points obtained from (a).

Because of the excellent PA performance and good biocompatibility of the BSA-Boca-BODIPY NPs in vitro, the in vivo PA imaging was performed on a subcutaneous 4T1 breast cancer tumor-bearing mouse model at the tumor size reached to ~100 mm^3^, which was anesthetized with isoflurane (**Figure 5**B). The PA images of tumor cross section were recorded by the home-made PA tomography system at different time intervals. As demonstrated in Figure 5A, a very weak PA signal can be observed under the excitation at 800 nm before intravenous injection of the BSA-Boca-BODIPY NPs, which may ascribe to the hemoglobin endogenous contrast in blood vessels. An observable PA contrast enhancement in the tumor location was found after intravenous administration of the BSA-Boca-BODIPY NPs for 2 h. The PA signals were increased with the time, suggesting that the BSA-Boca-BODIPY NPs were able to accumulate in the tumor via enhanced permeability and retention (EPR) effect. Moreover, the signals were decreased after 10 h, indicating the BSA-Boca-BODIPY NPs can be metabolized from the mice. Furthermore, Quantitative PA signals exhibited that the intensity of the tumor at 10 h post-injection was about fourteen times higher than that before injection of the contrast agent (Figure 5C). To measure the biodistribution of the as-prepared NPs, ICG-conjugated BSA-Boca-BODIPY NPs (1 mg ICG/kg) was intravenously injected into tumor-bearing mice through tail-vein, and then FL images at different time points were performed using the in vivo FL imaging system. As shown in **Figure S27A**, the fluorescence signal of the ICG-conjugated BSA-Boca-BODIPY NPs was observed throughout the body at 6 h post-injection. And the fluorescence signal of tumor was gradually increased at 12 h post-injection, demonstrating the NPs can be accumulated in the tumor. At 24 h post-injection, the fluorescence signal was decreased, indicating the NPs was metabolized from the mice. The ex vivo imaging of tumor and major organs at 12 h post-injection was also obtained (**Figure S**27B), the signal can be mainly found in the liver, kidney, and tumor.

### In Vivo Photothermal Therapy

**Figure 6:**
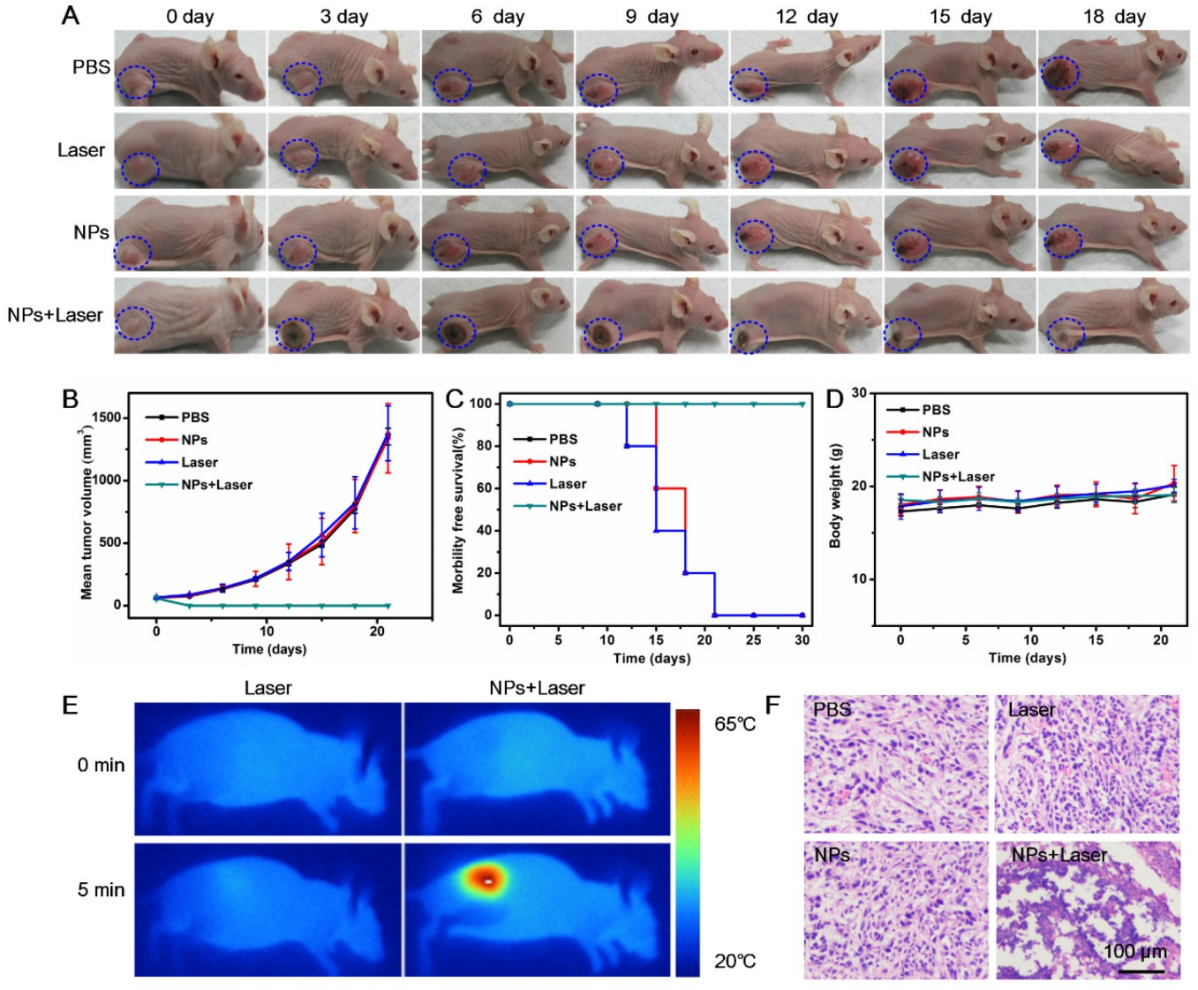
In vivo cancer PTT on 4T1 tumor-bearing nude mice using BSA-Boca-BODIPY NPs. A) Representative photos of 4T1 tumor-bearing mice at different days after different treatments, blue circle indicated the tumor sites; B) Tumor volumes, C) Survival rates, and D) Body weights of different groups of 4T1 tumor-bearing mice; E) Thermographic images of 4T1 tumor-bearing mice exposed to NIR laser for 5 min (808 nm, 0.75 w cm^-2^) with or without injection of BSA-Boca-BODIPY NPs; F) H&E staining images of tumor sections collected from 4T1 tumor-bearing mice with different treatments.

Due to the demonstrated PTT cytotoxicity in vitro and tumor accumulation through EPR effect of the BSA-Boca-BODIPY NPs, the in vivo PTT efficiency of the NPs under the irradiation of 808 nm NIR laser was further estimated with the use of subcutaneous 4T1 tumor-bearing nude mice. The mice involved in the experiment were divided randomly into four groups (five mice in each group) when the tumor size reached about 100 mm^3^. According to the results demonstrated in Figure 5, the BSA-Boca-BODIPY NPs can be accumulated in the tumor at a high concentration after 10 h intravenous injection. Therefore, 10 h post-injection was chosen to carry out the in vivo treatment. The temperature variations of the groups under irradiation of 808 nm laser were recorded through an infrared thermal camera. It can be observed that the temperature of the tumor site was nearly no change under laser irradiation alone, suggesting it can't generate hyperthermia in the tumor region. While the temperature of BSA-Boca-BODIPY NPs-mediated PTT treatment group was dramatically increased over 42 °C in 5 min after the laser irradiation, leading to tissue damage (**Figure** 6E). The results further demonstrated the tumor accumulation and strong in vivo photothermal efficiency of the BSA-Boca-BODIPY NPs. To assess the PTT efficiency of the as-prepared BSA-Boca-BODIPY NPs, the representative images and quantitative data of 4T1 tumor-bearing mice were obtained every 3 days after different treatments. As displayed in Figure 6A, the control groups including PBS-treated, Laser-treated, and BSA-Boca-BODIPY NPs-treated exhibited rapid tumor growth. The tumor size was greatly increased (Figure 6B), indicating that neither BSA-Boca-BODIPY NPs injection alone nor laser irradiation alone can suppress the tumor growth. It can also be observed that the tumor festered after 9 days and the area was enlarged with the time increasing. In contrast, the growth of tumor in the group with BSA-Boca-BODIPY NPs injection under the 808 nm laser irradiation was dramatically inhibited. Black scars at the original tumor location can be observed after the laser irradiation, which were disappeared two weeks later. Further, the survival rate of the 4T1 tumor-bearing mice was recorded after different treatment (Figure 6C). The subcutaneous tumor-bearing mice treated with PBS and laser alone started to die after 12 days and the phenomenon appeared in the NPs-treated group on day 15. On the contrary, the group treated with BSA-Boca-BODIPY NPs plus 808 nm laser irradiation exhibited a 100% survival after 30 days and no tumor recurrence was observed after the PTT treatment. Moreover, no obvious body weight variations of the mice were found among the four groups with different treatments (Figure 6D). It demonstrated there were nearly no side effects of the PTT treatment process. Furthermore, hematoxylin and eosin (H&E) staining analysis after different treatment was carried out. As shown in Figure 6F, the groups of PBS-treated, Laser-treated alone, and BSA-Boca-BODIPY NPs-treated alone had no effect on the tumor growth, while the NPs plus laser irradiation damage the tumor issues through cell necrosis. The results confirmed the as-obtained BSA-Boca-BODIPY NPs were excellent photothermal agents for destroying tumor tissues in vivo, which were consistent with the in vitro results.

### Histological Analysis

**Figure 7:**
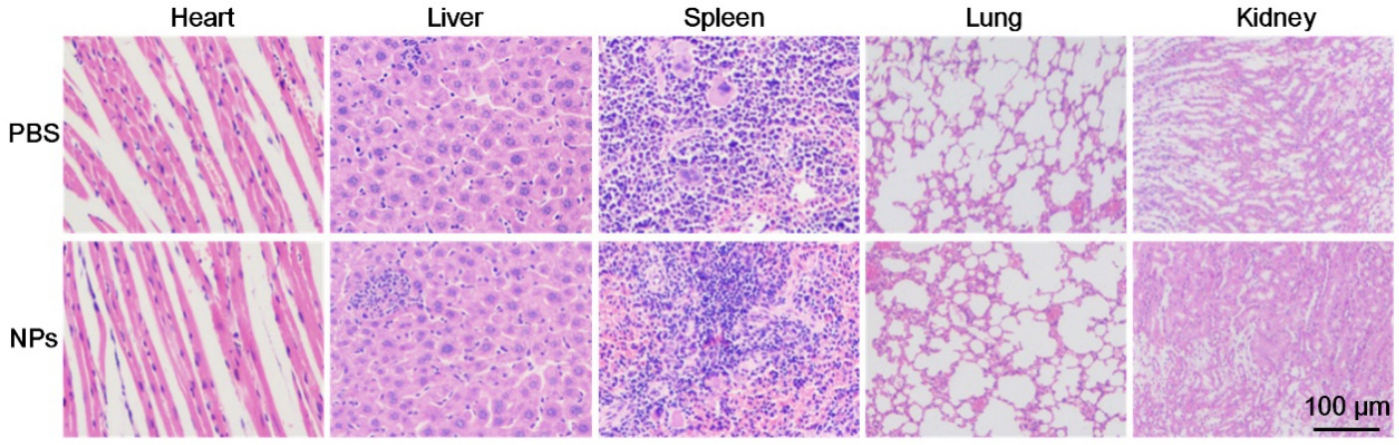
Representative H&E-stained images of major organs including the heart, liver, spleen, lung, and kidney collected from mice sacrificed 7 d after injection of PBS and BSA-Boca-BODIPY NPs, respectively.

For further potential clinical applications, histology analysis was carried out to evaluate the in vivo toxicity of the as-prepared BSA-Boca-BODIPY NPs. H&E stained images of the major organ slices (heart, liver, spleen, lung, and kidney) treated with PBS or BSA-Boca-BODIPY NPs were obtained. As displayed in **Figure 7**, the images didn't exhibit any obvious morphology damage or inflammation in the two groups, indicating the excellent histocompatibility of the BSA-Boca-BODIPY NPs. The results also proved the BSA-Boca-BODIPY NPs were adequate for applying in the biological field.

## Conclusions

In summary, a small organic Boca-BODIPY molecule with strong NIR absorption was synthesized through molecule engineering which was introduced heavy atoms and alkyl chains to enhance the photothermal efficiency and the interaction with BSA. The water-dispersible biocompatible Boca-BODIPY-based NPs were then constructed in assistant of the BSA by self-assembly method for PA imaging-guided PTT applications. As expected, the as-prepared BSA-Boca-BODIPY NPs exhibited extensive PA signals and high photothermal conversion efficiency since the inhibited fluorescence and ROS generation ability through molecule engineering. The BSA-Boca-BODIPY NPs exhibited stabilities in terms of storage, pH, oxidation as well as photothermal stability, which was better than that of the FDA-approved ICG molecules. In vitro cytotoxicity experiments validated that the BSA-Boca-BODIPY NPs had no effect on the cell viability without laser irradiation. On the contrary, the NPs manifested the photothermal ability to destroy cancer cells under the NIR laser irradiation. Further, in vivo PA imaging results indicated the BSA-Boca-BODIPY NPs were effective contrast agents for tumor imaging, which can accumulate in the tumor through EPR effect and metabolize from the living system after 10 h. Moreover, Our NPs were proved to be an excellent candidate for photothermal tumor therapy through their amazing performance in tumor inhibition, which was confirmed not only by the tumor growth curve but also by histological staining of tumor slices. More importantly, the BSA-Boca-BODIPY NPs also exhibited good histocompatibility. Therefore, excellent biocompatible theranostic agents were supplied for PA imaging-guided photothermal tumor therapy. The study also expanded the applications of BODIPY molecules which would open a door to develop more useful NIR-absorbing organic small molecules for in vivo imaging-guided cancer therapy.

## Supplementary Material

Supplementary ^1^H NMR Specta and^ 13^C NMR Spectra of the related orgnaic molecules, fluorescence, stability, photothermal conversion efficiency of the BSA-Boca-BODIPY NPs.

## Figures and Tables

**Table 1:** Comparison of the photothermal conversion efficiency of different NPs

NPs	Photothermal conversion efficiency	Laser (nm)	Ref.
Ce6-labeled CuS NPs	29.8%	1064	62
Dopmine-melanin NPs	40%	808	63
BT-BIBDF Pdots	34.70%	785	64
Co-loaded CPNs	47.6%	808	65
DPP-DT-H Pdots	55%	770	66
Commercial Au nanorods	21%	800	60
BSA-Boca-BODIPY NPs	58.7%	808	this work

## Abbreviations

BODIPY: boron-dipyrromethene
Boca-BODIPY: B, O-chelated BODIPY
BSA: bovine serum albumin
PTT: photothermal therapy
NIR: near-infrared
PDT: photodynamic therapy
ROS: reactive oxygen species
