# Supplementary material for: Molecular Engineering of Near-Infrared Light-Responsive BODIPY-Based Nanoparticles with Enhanced Photothermal and Photoacoustic Efficiencies for Cancer Theranostics
Source: Theranostics. 2019 Jul 9;9(18):5315–31. doi: 10.7150/thno.34418 (PMC6691584; doi:10.7150/thno.34418)
Supplement: Supplementary file 1 — Supplementary 1H NMR Specta and 13C NMR Spectra of the related orgnaic molecules, fluorescence, stability, photothermal conversion efficiency of the BSA-Boca-BODIPY NPs. [file thnov09p5315s1.pdf]

Supplementary Material

Molecular Engineering of Near-Infrared Light-Responsive BODIPY-Based Nanoparticles with Enhanced Photothermal and Photoacoustic Efficiencies for Cancer Theranostics

Duyang Gao, [a,b#] *Boyu Zhang,*[c,a#] *Yubin Liu,*[a] *Dehong Hu,*[b] *Zonghai Sheng,*[b]* *Xuanjun Zhang,*[a]* *Zhen Yuan*[a]*

a. Faculty of Health Sciences, University of Macau, Macau SAR, 999078, PR China

b. Paul C. Lauterbur Research Center for Biomedical Imaging, Institute of Biomedical and Health Engineering, Shenzhen Institutes of Advanced Technology, Chinese Academy of Sciences, Shenzhen, 518055, China.

c. College of Medical Laboratory, Dalian Medical University, No. 9 West Section LvShun South Road, Dalian 116044, P. R. China

# Duyang Gao and Boyu Zhang contributed equally to this work.

*Corresponding authors: Z. Yuan (zhenyuan@umac.mo), X. J. Zheng (xuanjunzhang@umac.mo), Z. H. Sheng (zh.sheng@siat.cn)

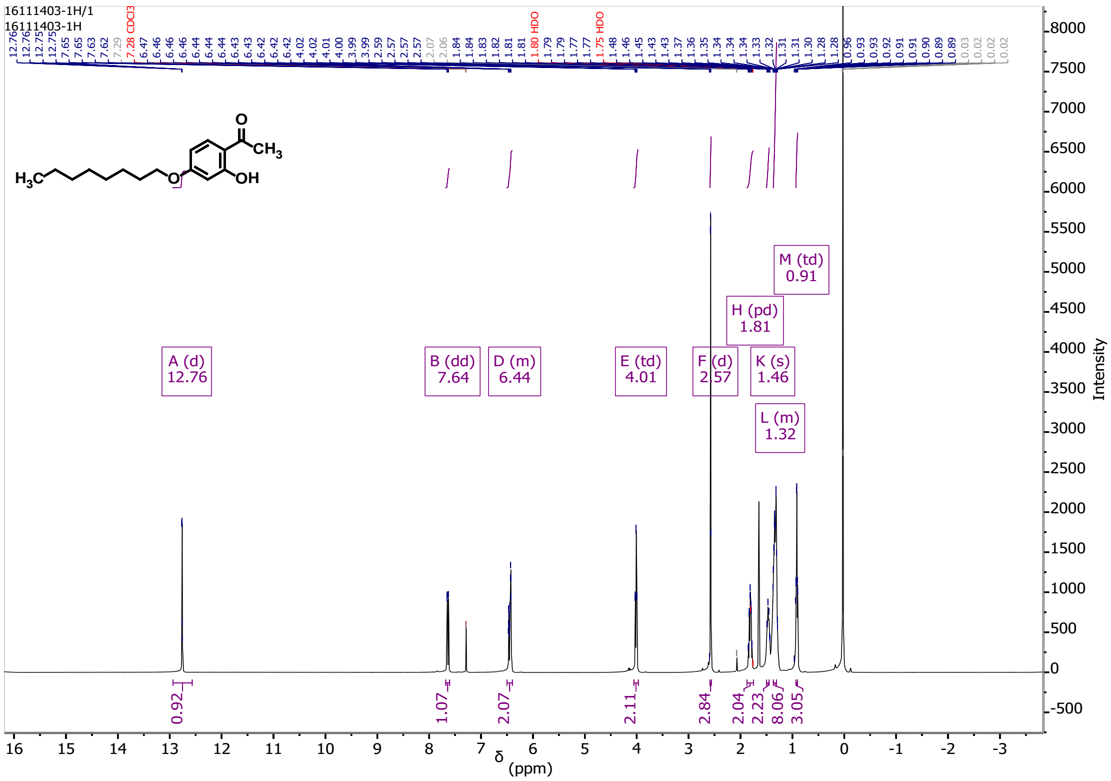

Figure S1. ^{1}H NMR Spectrum of 1-(2-hydroxy-4-(octyloxy)phenyl)ethan-1-one (**1**)

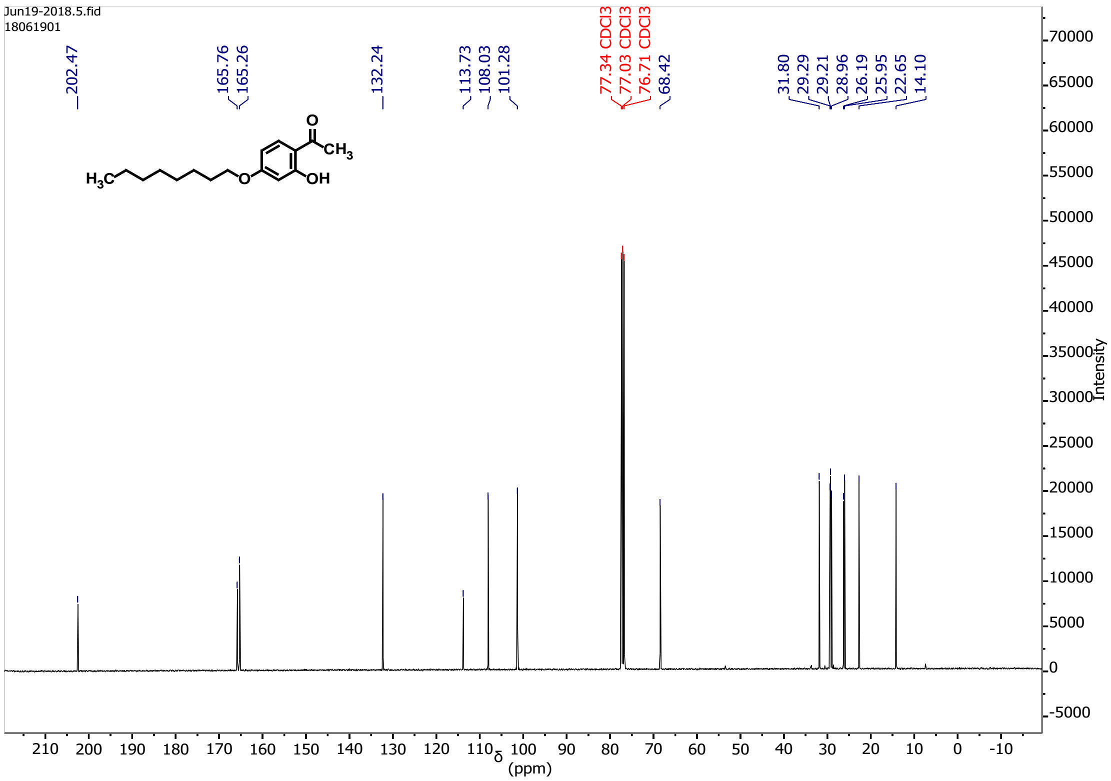

Figure S2. ^{13}C NMR Spectrum of 1-(2-hydroxy-4-(octyloxy)phenyl)ethan-1-one (**1**)

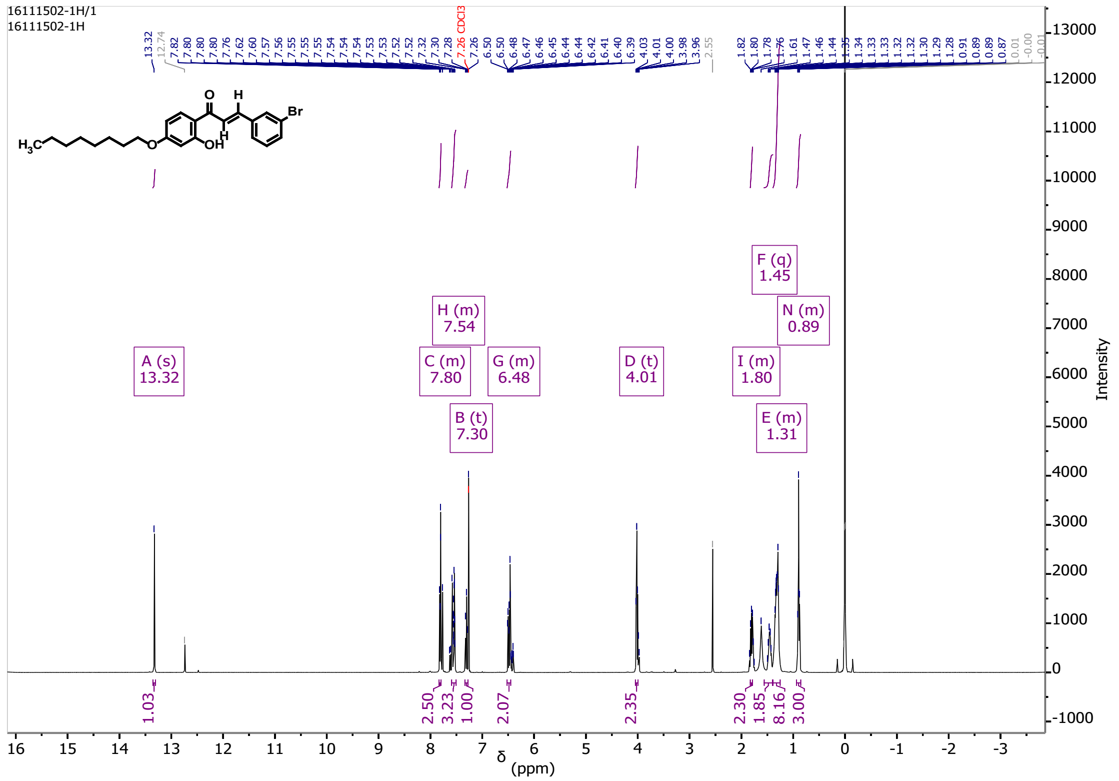

Figure S3. ^{1}H NMR Spectrum of (E)-3-(3-bromophenyl)-1-(2-hydroxy-4-(octyloxy)phenyl)prop-2-en-1-one (**3**)

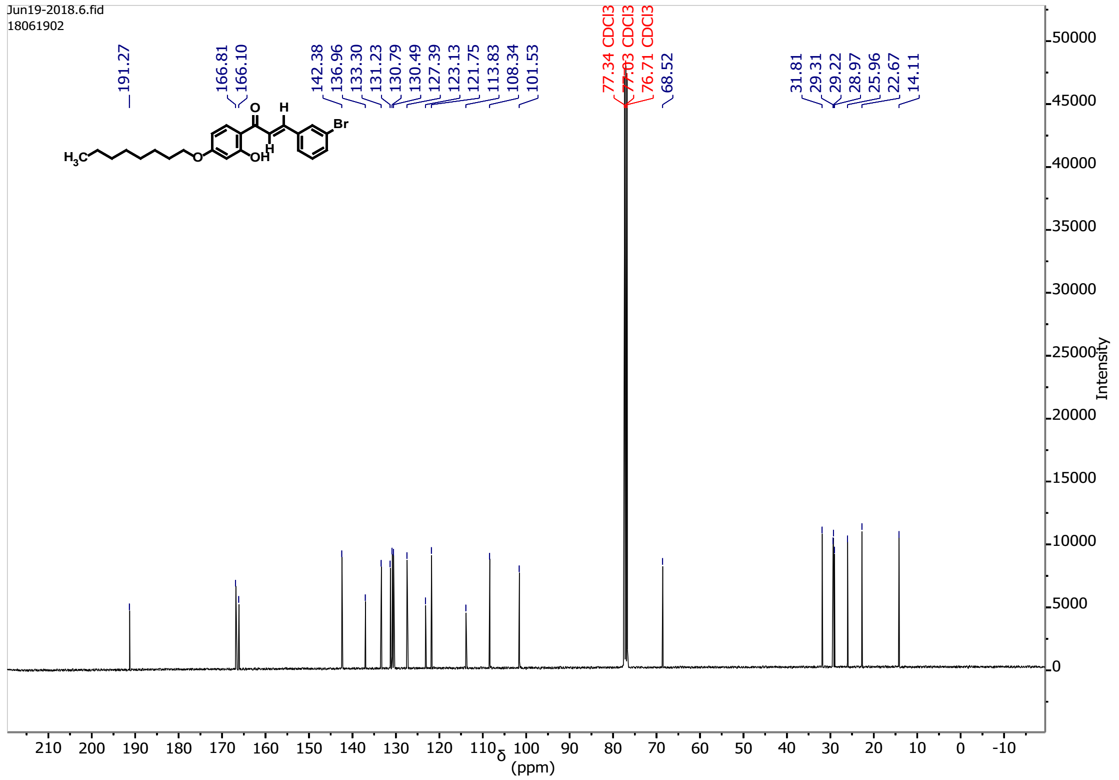

Figure S4. ^{13}C NMR Spectrum of (E)-3-(3-bromophenyl)-1-(2-hydroxy-4-(octyloxy)phenyl)prop-2-en-1-one (**3**)

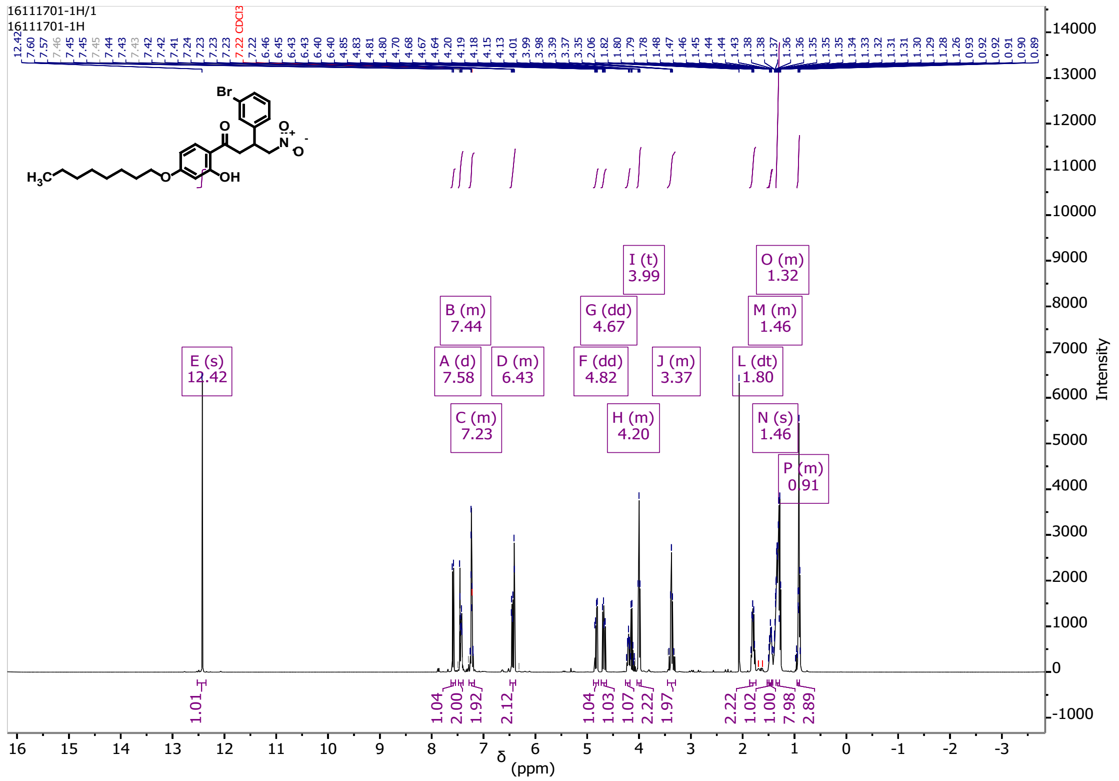

Figure S5. ^{1}H NMR Spectrum of (Z)-3-(3-bromophenyl)-1-(2-hydroxy-4-(octyloxy)phenyl)-4-nitrobut-2-en-1-one (**4**)

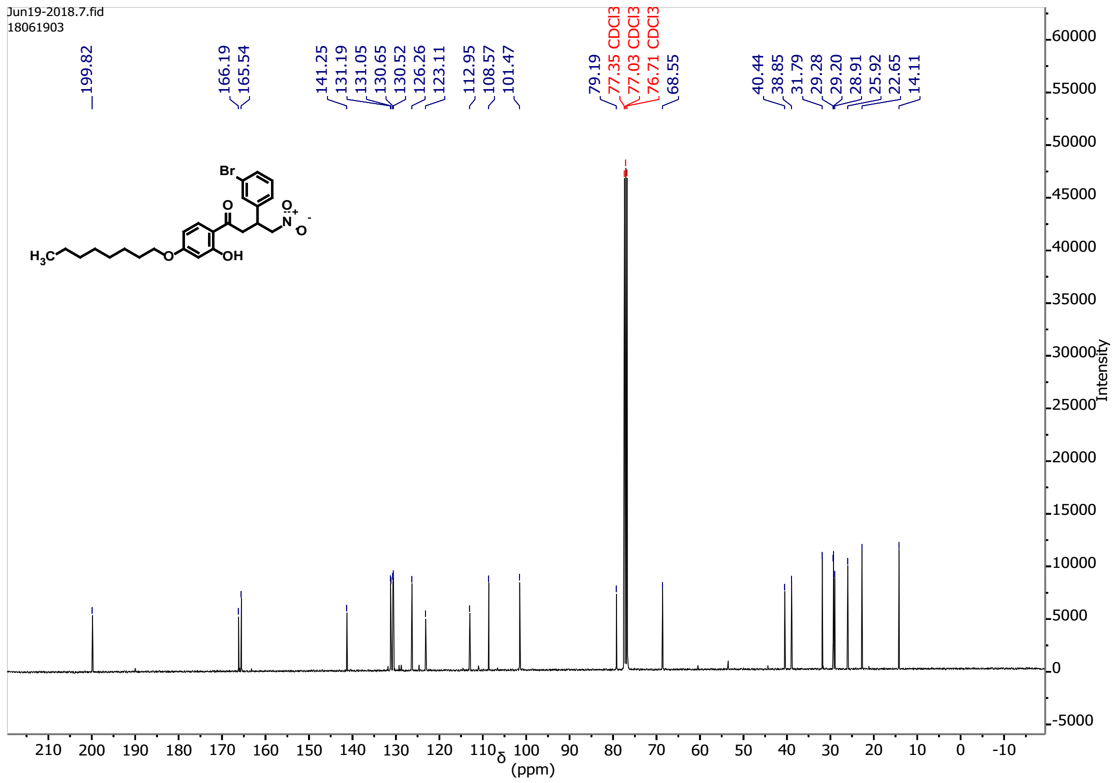

Figure S6. ^{13}C NMR Spectrum of (Z)-3-(3-bromophenyl)-1-(2-hydroxy-4-(octyloxy)phenyl)-4-nitrobut-2-en-1-one (**4**)

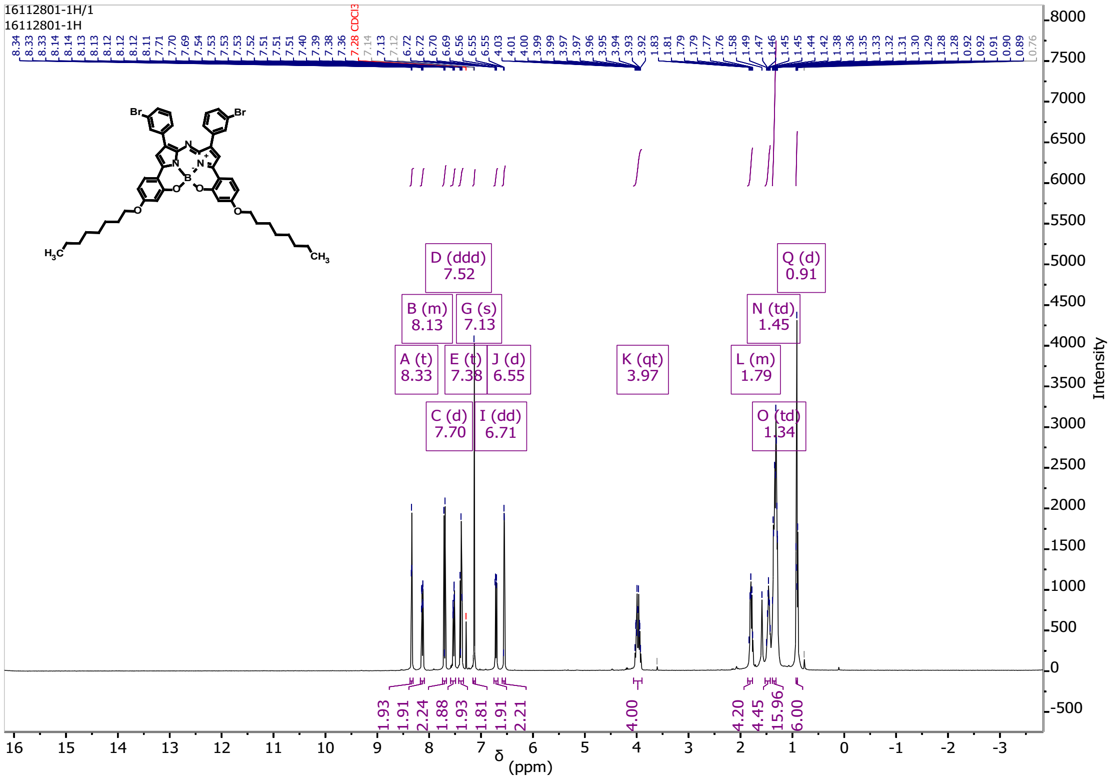

Figure S7. ^{1}H NMR Spectrum of Boca-BODIPY

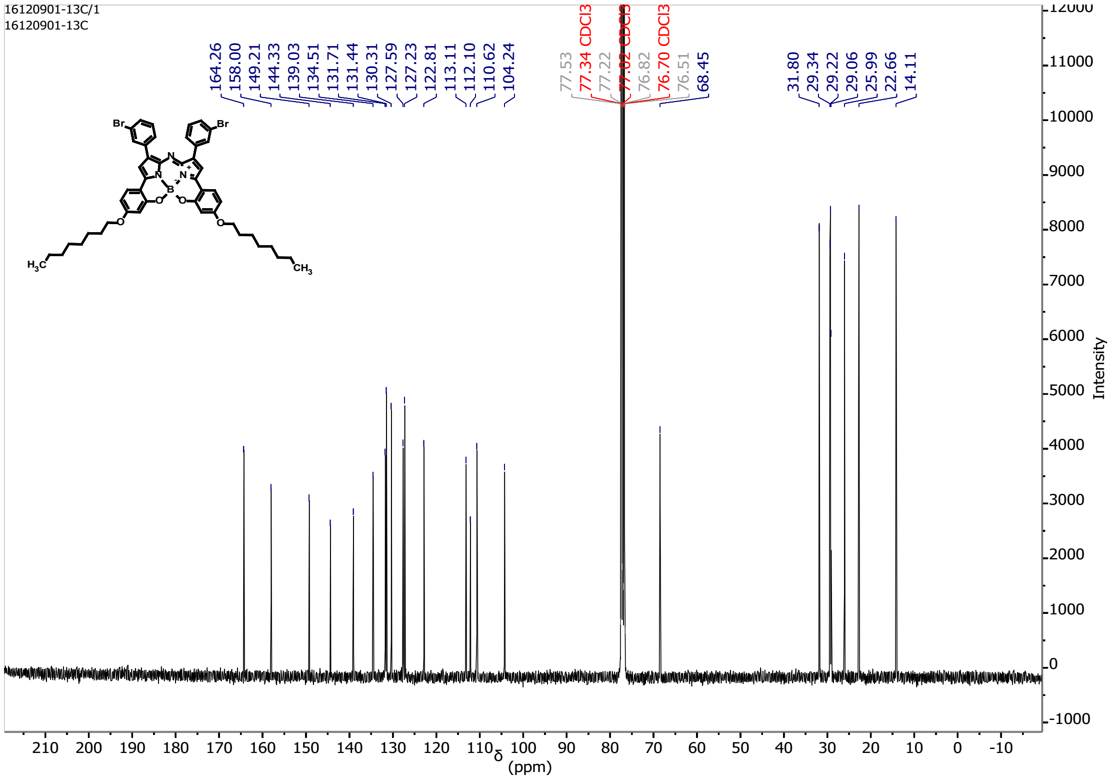

Figure S8. ^{13}C NMR Spectrum of Boca-BODIPY

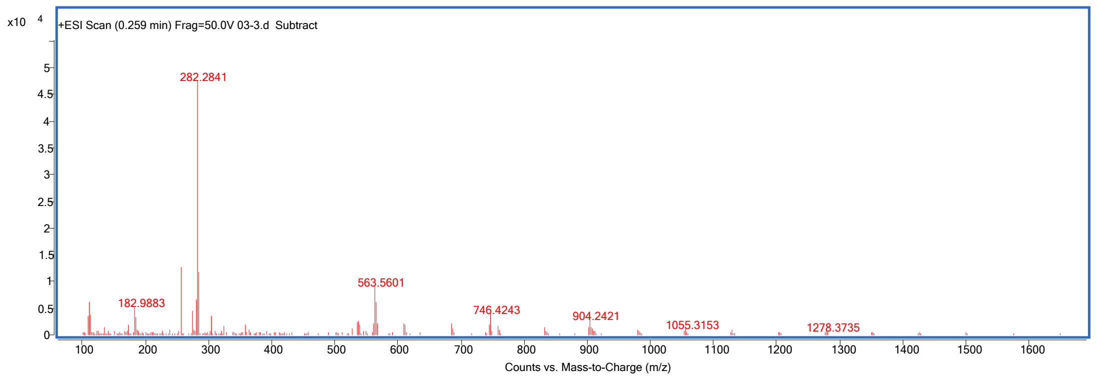

Figure S9. HRMS-ESI spectrum of Boca-BODIPY

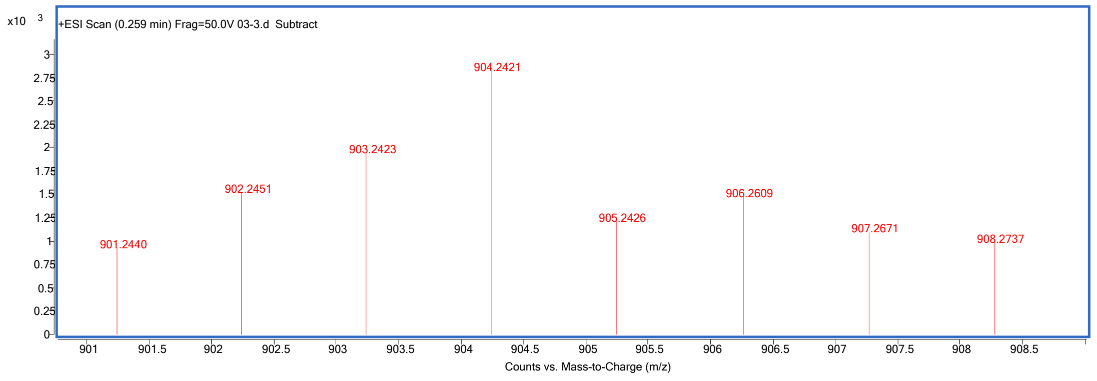

Figure S10. Isotope peaks of Boca-BODIPY's molecular ion peak

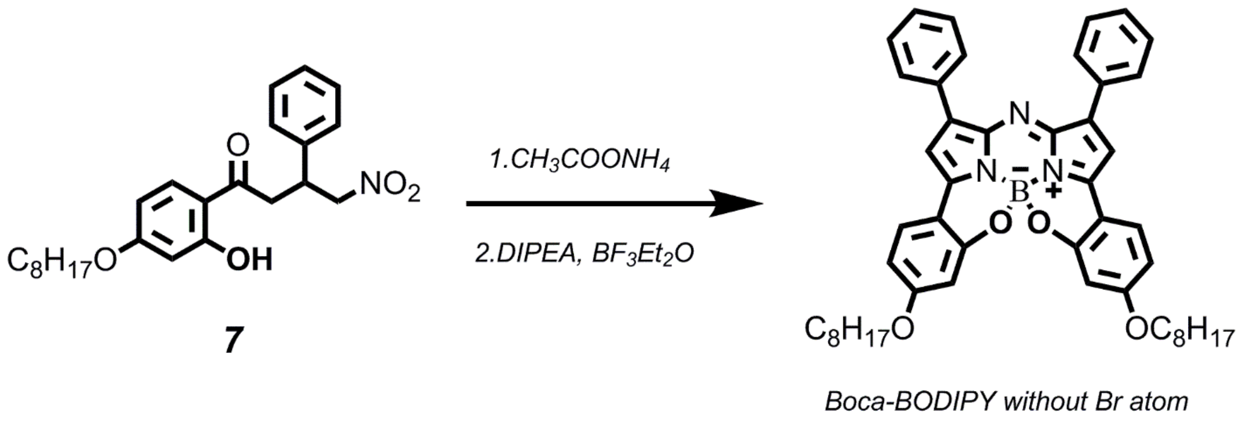

Scheme S1. Synthetic route to compound Boca-BODIPY without Br atom.

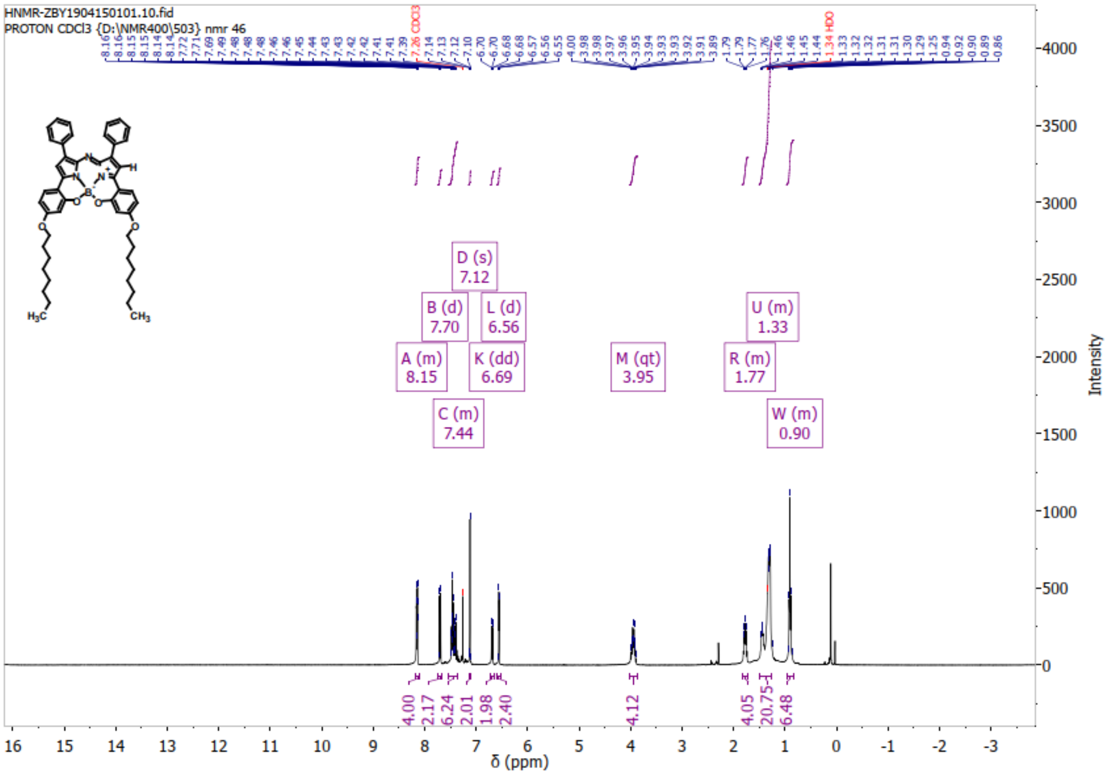

Figure S11. ^{1}H NMR Spectrum of Boca-BODIPY molecule without Br atom.

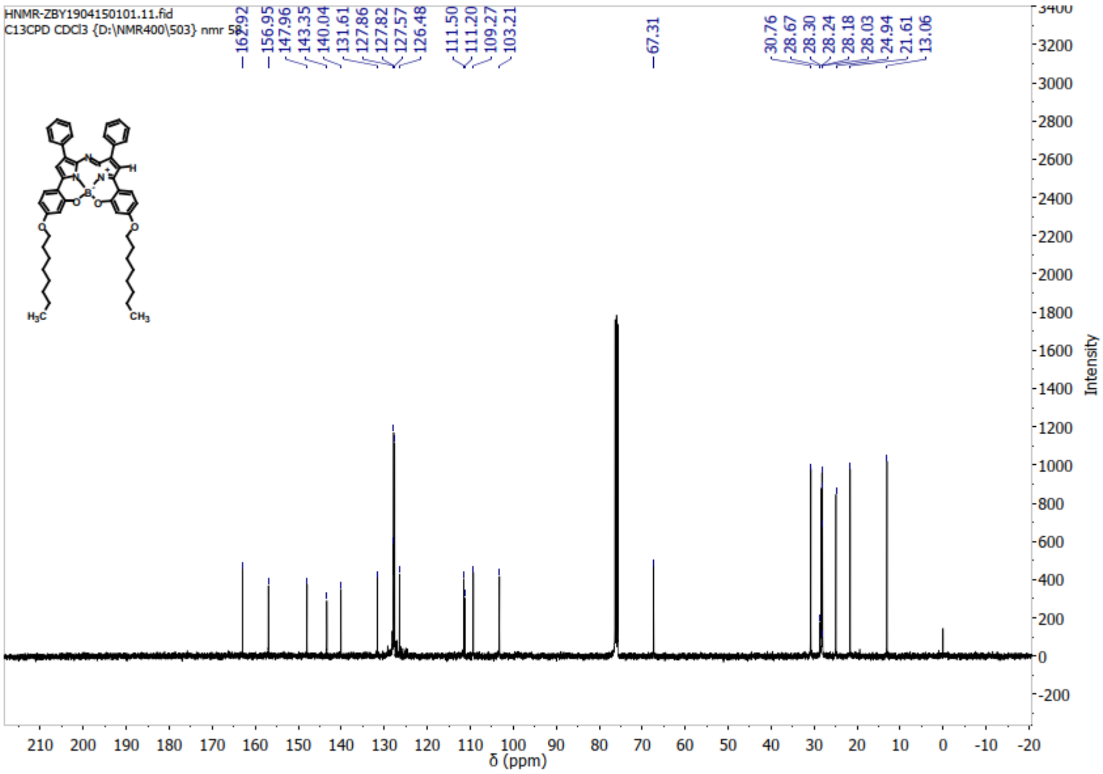

Figure S12. ^{13}C NMR Spectrum of Boca-BODIPY molecule without Br atom.

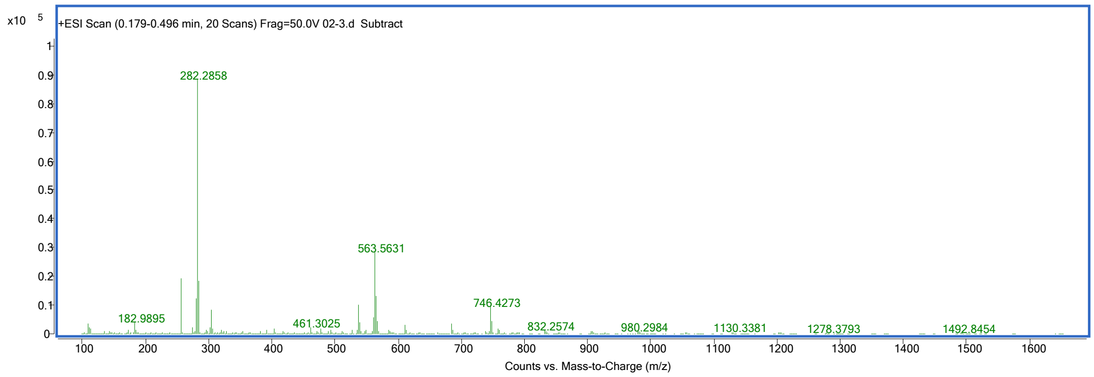

Figure S13. HRMS-ESI spectrum of Boca-BODIPY molecule without Br atom.

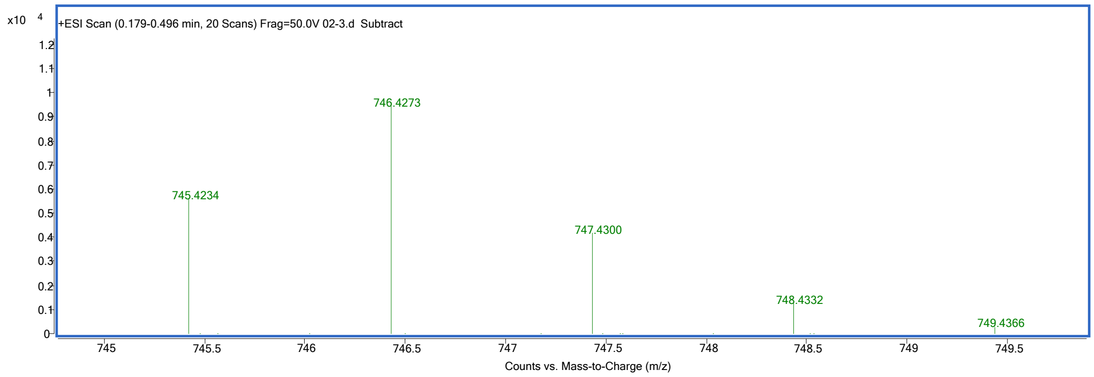

Figure S14. Isotope peaks of Boca-BODIPY molecule without Br atom.

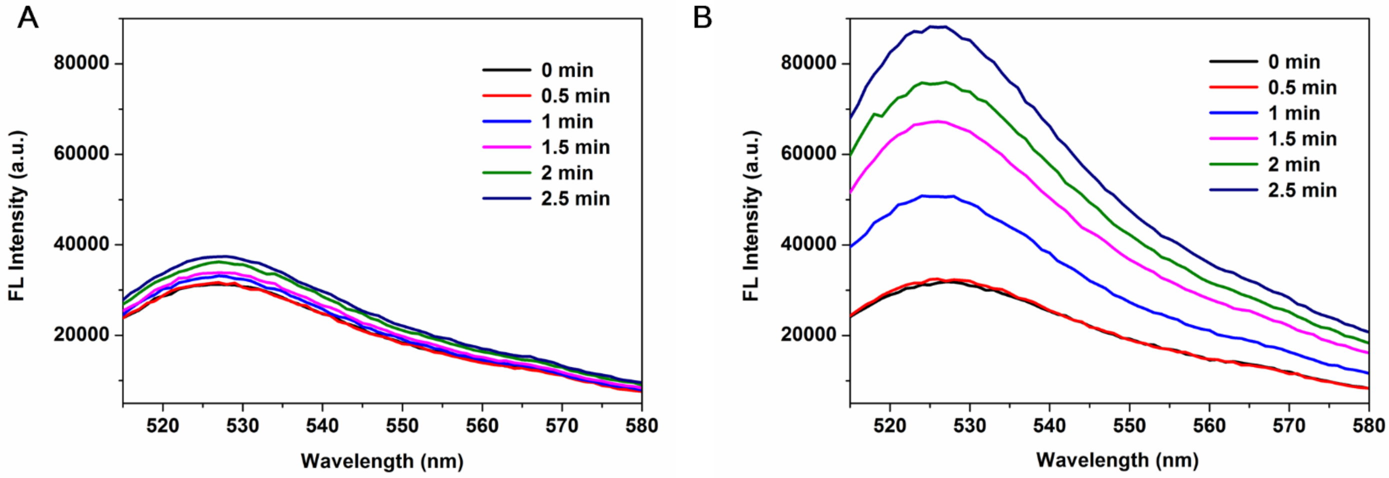

Figure S15. Fluorescence intensity of the singlet oxygen sensor green (SOSG) under the irradiation of NIR laser at the same concentration of A) Boca-BODIPY molecules and B) Boca-BODIPY molecules without Br atoms.

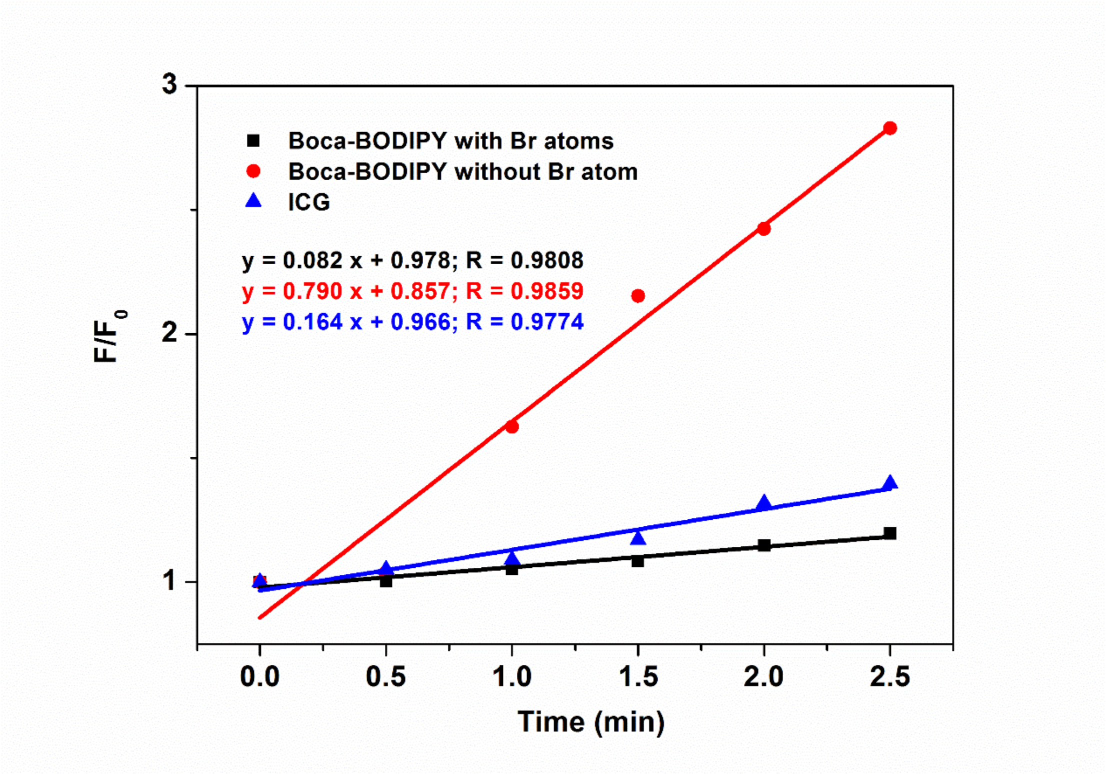

Figure S16. Fluorescence intensity variations of SOSG with presence of ICG, Boca-BODIPY molecule with or without Br atoms versus laser irradiation time.

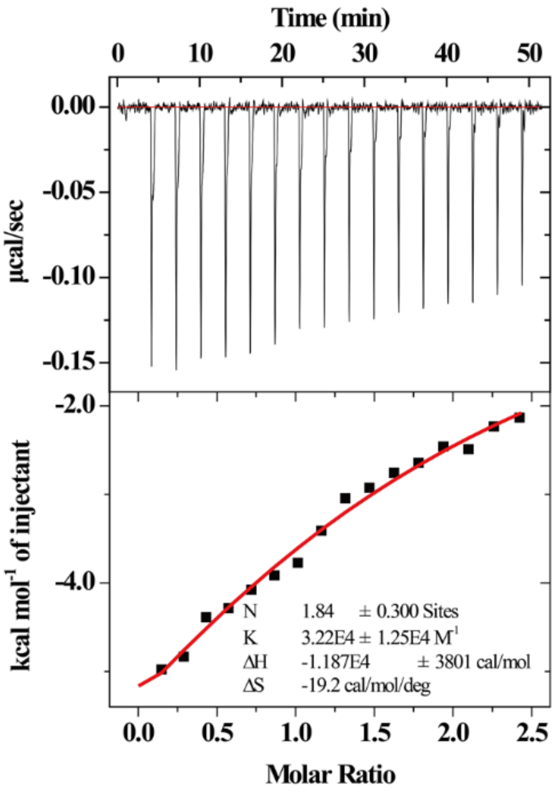

Figure S17. Characterization of the binding between BSA and Boca-BODIPY using ITC.

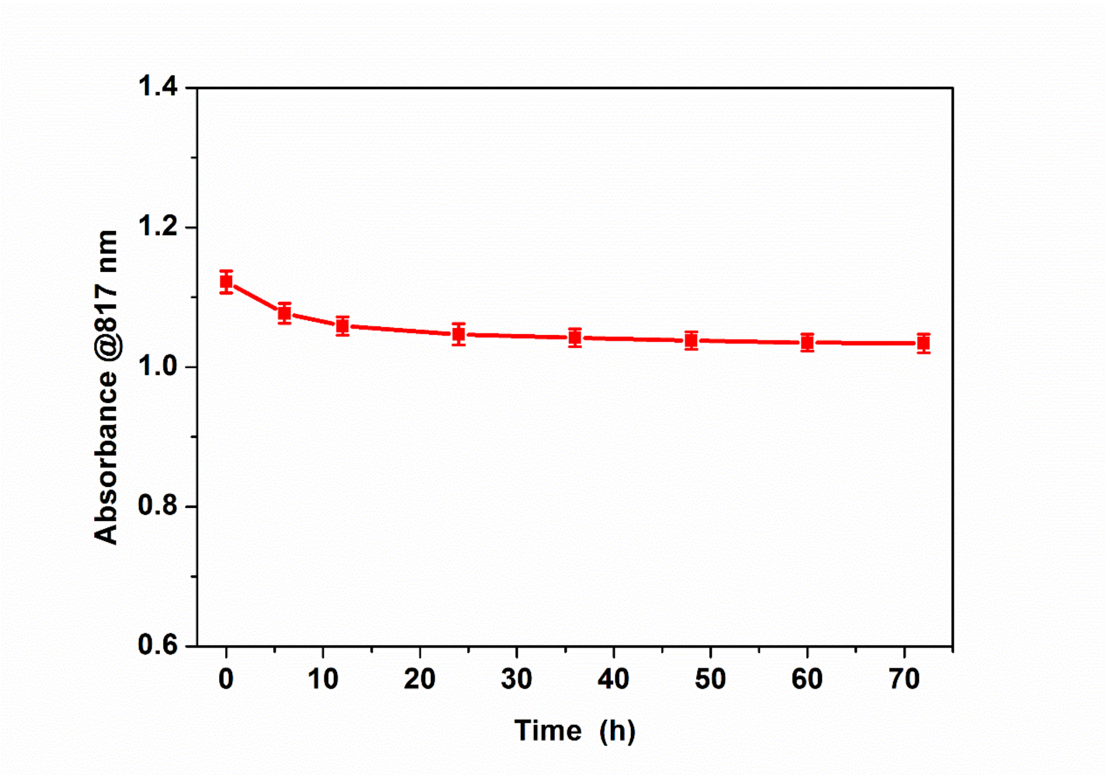

Figure S18. Absorbance at 817 nm of the BSA-Boca-BODIPY nanoparticles at different time points.

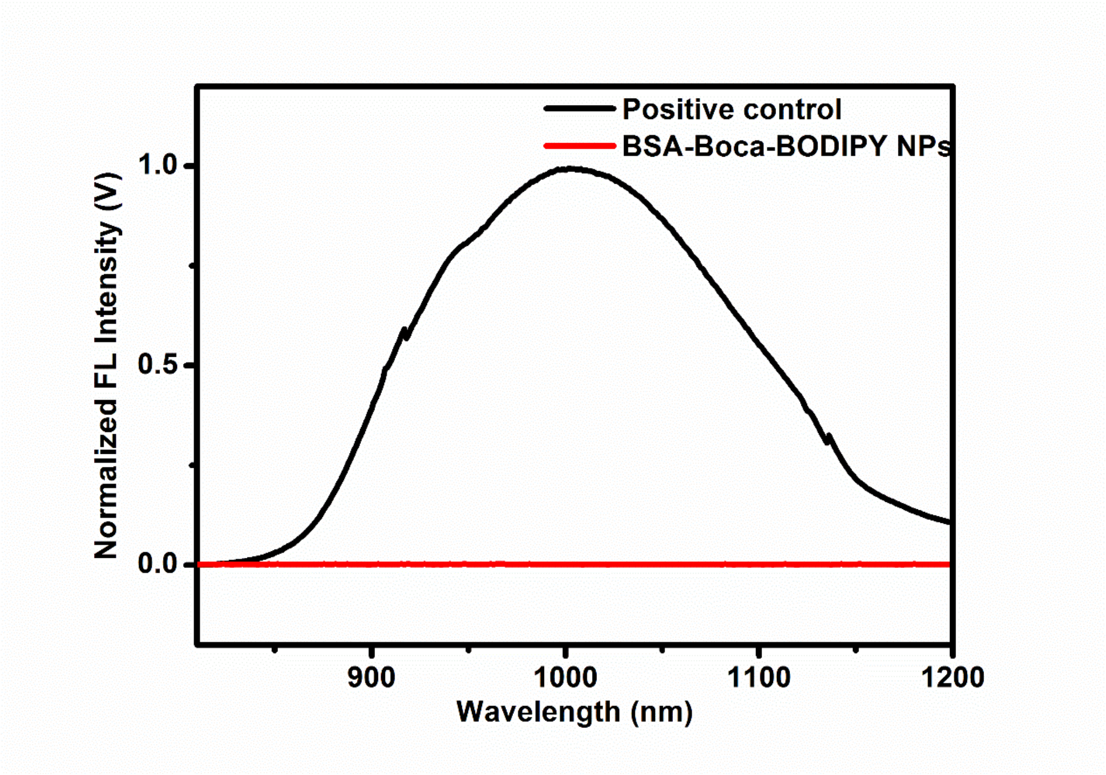

Figure S19. Fluorescence of BSA-Boca-BODIPY NPs and a positive control under 800 nm excitation.

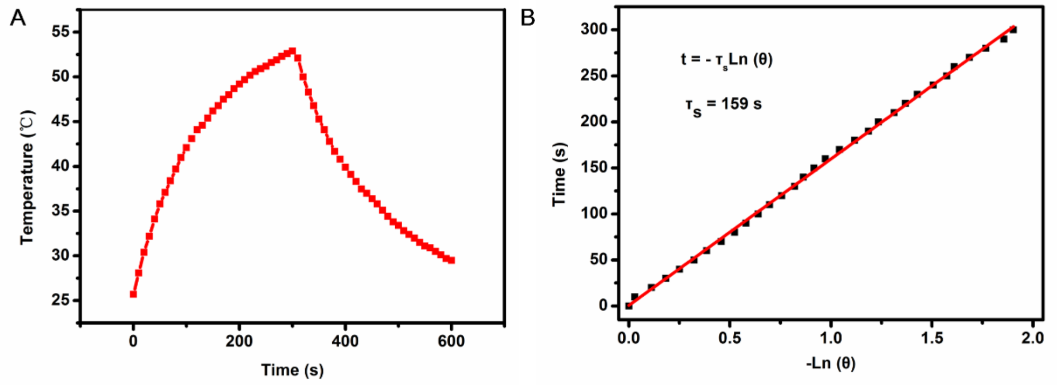

Figure S20. A) BSA-Boca-BODIPY NPs solution (50 µg mL^{-1}) under laser irradiation at a wavelength of 808 nm (0.75 w cm^{-2}) for 300 s and the laser was turned off after irradiation; A) plot of cooling period versus negative natural logarithm of the temperature driving force obtained from the cooling stage as displayed in A). The time constant for heat transfer of the system is determined to be τ_s = 159 s.

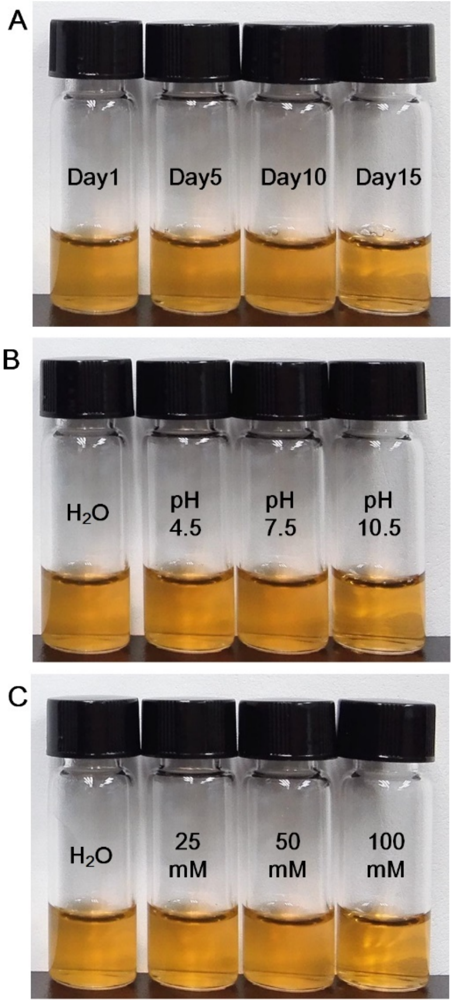

Figure S21. Stability of the BSA-Boca-BODIPY NPs. A) Photographs of the as-prepared BSA-Boca-BODIPY NPs dispersed in water for two weeks, B) Photographs of the as-prepared BSA-Boca-BODIPY NPs dispersed in PBS with different pH values, C) Photographs of the as-prepared BSA-Boca-BODIPY NPs dispersed in different concentrations of H_2O_2.

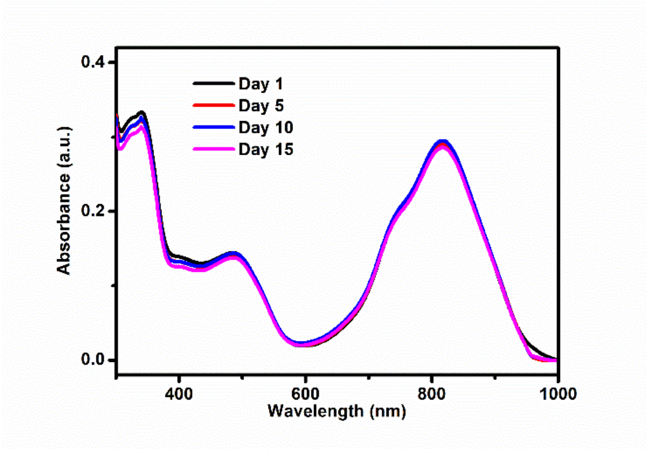

Figure S22. Absorbance of the BSA-Boca-BODIPY NPs at different time points.

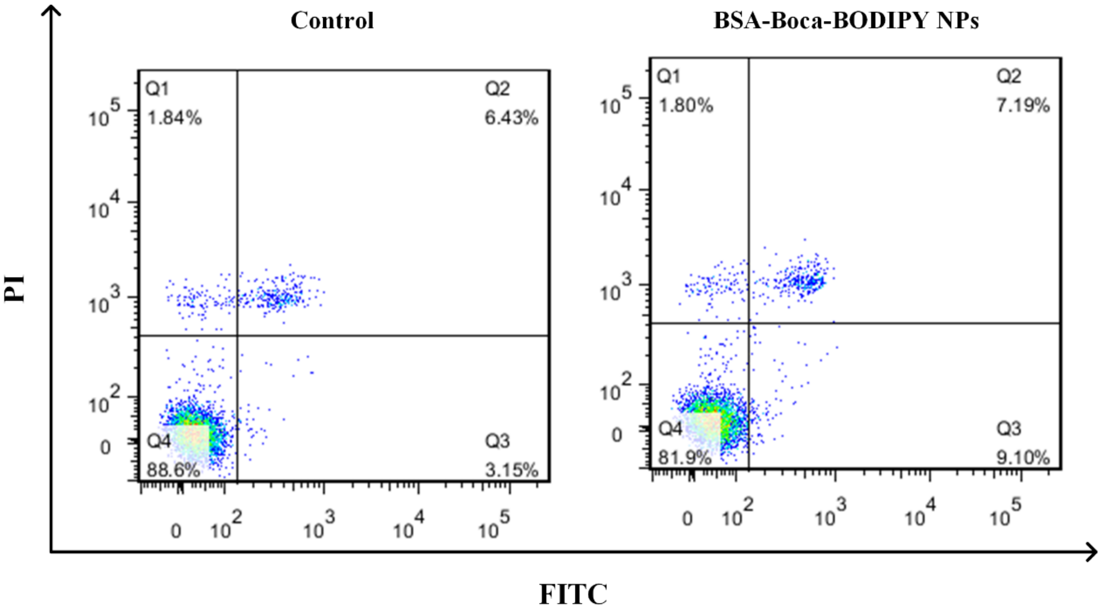

Figure S23. Measurement of BSA-Boca-BODIPY NPs-induced cell apoptosis using flow cytometry.

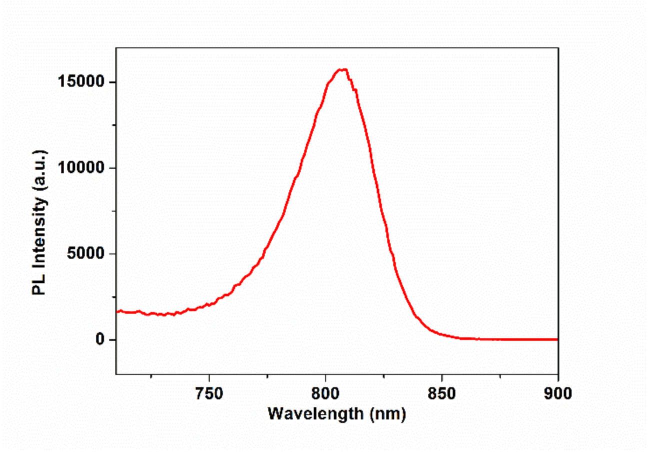

Figure S24. Fluorescence emission of the ICG-conjugated BSA-Boca-BODIPY NPs.

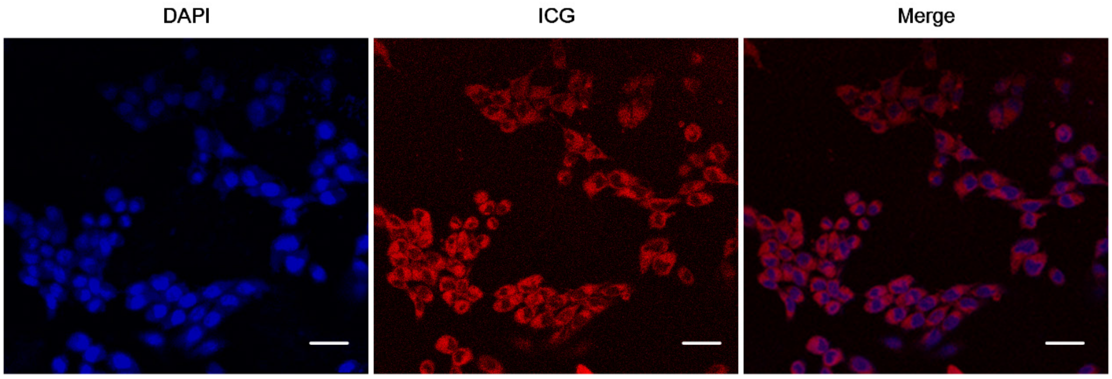

Figure S25. Confocal fluorescence images of 4T1 cells with ICG-conjugated BSA-Boca-BODIPY nanoparticles after 12 h incubation. Blue represented DAPI fluorescence and red showed fluorescence of ICG-conjugated BSA-Boca-BODIPY nanoparticles, scale bar was 25 μm.

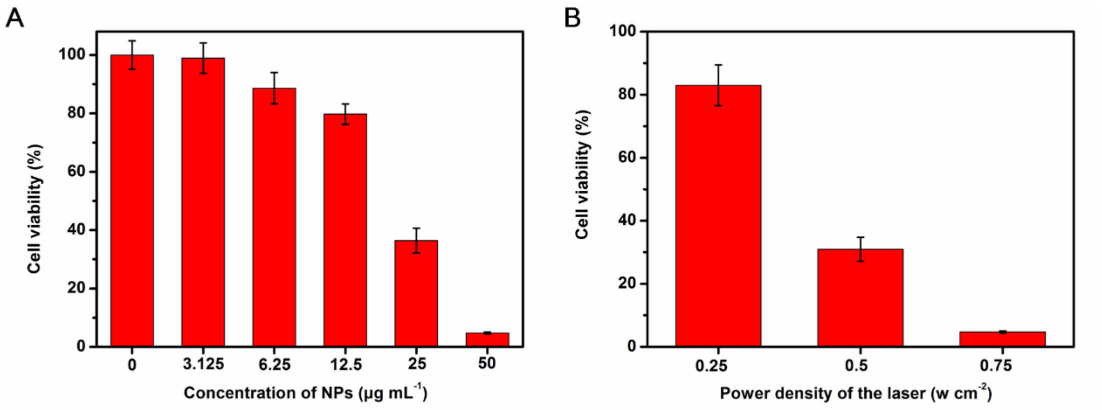

Figure S26. Cell viabilities of 4T1 breast cancer cells treated with A) different concentrations of BSA-Boca-BODIPY NPs under the irradiation of laser (0.75 w cm^{-2}, 5 min) and B) different power density of laser at the same concentration of BSA-Boca-BODIPY NPs (50 μg mL^{-1}).

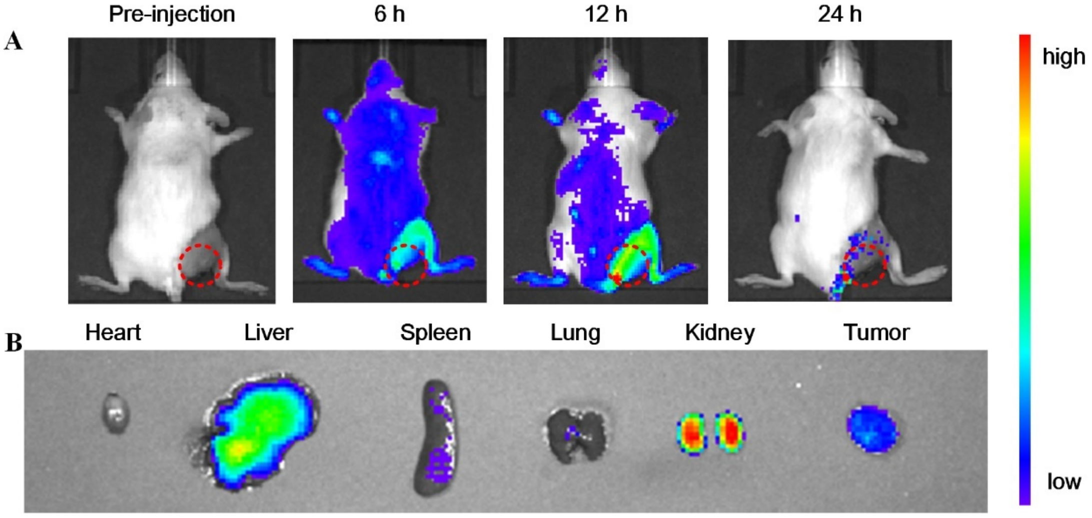

Figure S27. In vivo biodistribution of the BSA-Boca-BODIPY NPs. Red circle indicated the tumor.
